# IGF-1 receptor antagonism inhibits autophagy

**DOI:** 10.1093/hmg/ddt300

**Published:** 2013-06-25

**Authors:** Maurizio Renna, Carla F. Bento, Angeleen Fleming, Fiona M. Menzies, Farah H. Siddiqi, Brinda Ravikumar, Claudia Puri, Moises Garcia-Arencibia, Oana Sadiq, Silvia Corrochano, Sarah Carter, Steve D.M. Brown, Abraham Acevedo-Arozena, David C. Rubinsztein

**Affiliations:** 1 Department of Medical Genetics, Cambridge Institute for Medical Research, University of Cambridge, Wellcome/MRC Building, Addenbrooke's Hospital, Hills Road, Cambridge CB2 0XY, UK; 2 Department of Physiology, Development and Neuroscience, University of Cambridge, Cambridge, UK; 3 Mammalian Genetics Unit, Medical Research Council, Harwell, UK

## Abstract

Inhibition of the insulin/insulin-like growth factor signalling pathway increases lifespan and protects against neurodegeneration in model organisms, and has been considered as a potential therapeutic target. This pathway is upstream of mTORC1, a negative regulator of autophagy. Thus, we expected autophagy to be activated by insulin-like growth factor-1 (IGF-1) inhibition, which could account for many of its beneficial effects. Paradoxically, we found that IGF-1 inhibition attenuates autophagosome formation. The reduced amount of autophagosomes present in IGF-1R depleted cells can be, at least in part, explained by a reduced formation of autophagosomal precursors at the plasma membrane. In particular, IGF-1R depletion inhibits mTORC2, which, in turn, reduces the activity of protein kinase C (PKCα/β). This perturbs the actin cytoskeleton dynamics and decreases the rate of clathrin-dependent endocytosis, which impacts autophagosome precursor formation. Finally, with important implications for human diseases, we demonstrate that pharmacological inhibition of the IGF-1R signalling cascade reduces autophagy also in zebrafish and mice models. The novel link we describe here has important consequences for the interpretation of genetic experiments in mammalian systems and for evaluating the potential of targeting the IGF-1R receptor or modulating its signalling through the downstream pathway for therapeutic purposes under clinically relevant conditions, such as neurodegenerative diseases, where autophagy stimulation is considered beneficial.

## INTRODUCTION

The insulin-like growth factor (IGF-1) binds to extracellular insulin-like growth factor receptors, such as the insulin-like growth factor-1 receptor (IGF-1R), resulting in their activation and phosphorylation. The tyrosine kinase activities of these receptors phosphorylate signalling molecules, including the insulin receptor substrate (IRS) protein family. Once phosphorylated, the IRS proteins act as molecular adaptors to facilitate downstream signalling pathways via AKT, which serves as a major effector. Inhibition of insulin/IGF-1 signalling is considered an exciting therapeutic target, as this modulation increases lifespan in worms, flies and mice, and also protects model organisms from neurodegenerative insults (1–4).

Autophagy, a critical cytoprotective pathway, involves the formation of double-membrane autophagosomes that capture portions of cytoplasm before fusing with lysosomes where their contents are ultimately degraded (5). Autophagy is a critical pathway that regulates the accumulation of such cytoplasmic aggregate-prone proteins, such as mutant huntingtin, associated with neurodegenerative diseases. Autophagy-inducing drugs and genes can alleviate the toxicity of mutant huntingtin and related proteins in cell and animal models of disease (6). Furthermore, autophagy up-regulation in model organisms increases longevity (7). In mammalian cells, autophagosomes are formed from precursor membrane structures that include a complex of the autophagic proteins Atg5, Atg12 and Atg16L1. These precursors have been proposed to originate from a variety of sources, including the plasma membrane, the endoplasmic reticulum and mitochondria (8). These sources may not be mutually exclusive; although the endoplasmic reticulum and mitochondria appear only to be relevant under starvation conditions when autophagy is induced, while the plasma membrane contributes to autophagosome precursors under both induced and basal autophagy conditions (9). The plasma membrane is recruited to autophagosome precursors as a result of Atg16L1 binding to clathrin-coated pits (9). After endocytosis, which is required for autophagosome formation, the autophagosome precursors undergo homotypic fusion, which increases their size. This appears to be a prerequisite for recruiting the protein LC3-II, and the transition to a phagophore, which starts to engulf cytoplasmic contents before its edges fuse to become a completed autophagosome (10). At this stage, the Atg5-12-16L1 complex dissociates from the autophagosome. LC3-II is the only known protein that specifically associates with autophagosomes and not with other vesicular structures (11), and a standard way of measuring autophagosome number is by assaying LC3-II levels (as a function of actin/tubulin) or by counting the number of LC3-positive vesicles (11).

It has been reported that both IGF-1 inhibition and autophagy activation have beneficial effects on lifespan (1–4) and neurodegenerative insults (6,7). Since IGF-1 has been reported to block autophagy through AKT inhibition, which would be expected to occur via AKT activation of the direct autophagy inhibitor rapamycin complex 1 (mTORC1) (12,13), we anticipated chronic IGF-1 pathway inhibition to induce autophagy, which could account for many of its beneficial properties.

## RESULTS

### IGF-1R depletion decreases autophagy

**Figure 1.**
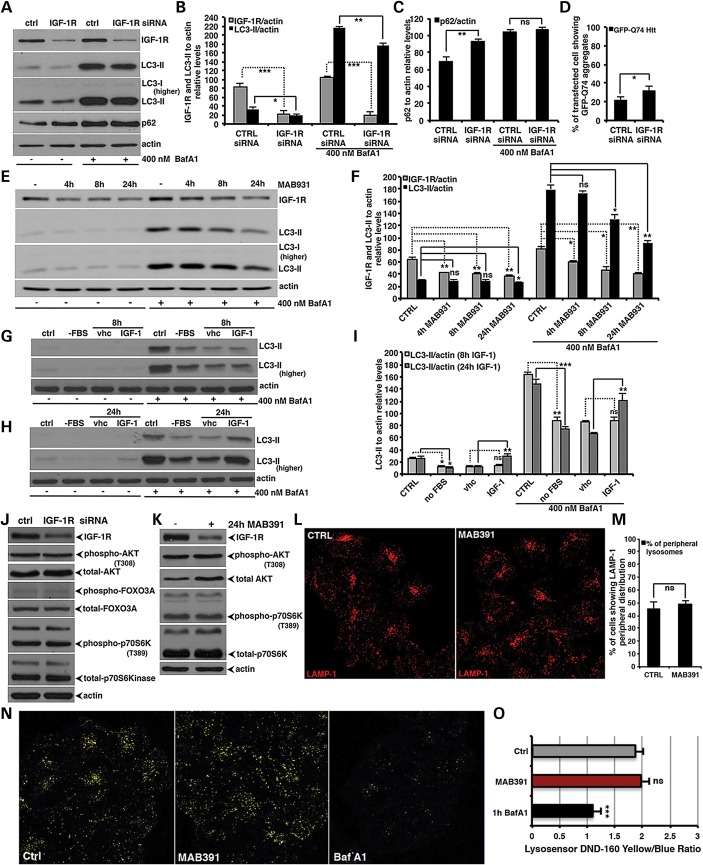
IGF1-R inhibition decreases autophagy. (**A–C**) HeLa cells were transfected for 72 h with 50 nm of either control or anti-IGF-1R siRNA. For the assessment of autophagy by LC3-II levels, bafilomycin A_1_ was added to the cells in the last 4 h before harvesting. The western blot panels are representative of at least three independent experiments performed in triplicate. The graphs in (B) and (C) report the IGF-1R, LC3-II and p62 levels relative to actin. The *P*-values were determined by two-tailed Student's *t*-test (B: *n* = 3; CTRL versus 24 h IGF-1R; *P = 0.0173; **P = 0.0048; C: *n* = 3; CTRL versus IGF-1R; **P = 0.0355). (**D**) HeLa cells were transfected for 96 h with 50nm of either control or anti-IGF-1R siRNA. In the last 48 h, cells were re-transfected with the same siRNA mixture plus the GFP-HD74 vector. The *P*-value for assessing the EGFP-HDQ74 aggregation was determined using Student's *t*-test (*n* = 3; CTRL versus IGF-1R siRNA, *P = 0.0283). (**E** and **F**) IGF1-R depletion time-dependently decreases autophagy. HeLa cells were treated for the indicated time points with the MAB391 neutralizing antibody. Bafilomycin A_1_ was added to the cells in the last 4 h. The graph reports IGF-1R and LC3-II levels relative to actin. The *P*-values were determined by Factorial Anova (*n* = 3; CTRL versus 24 h MAB391, *P = 0.0192 and **P = 0.0049). (**G** and **I**) HeLa cells were serum starved for 24 h and then stimulated or not (vhc, vehicle) by IGF-1 R_3_ for either 8 (G) or 24 h (H). Bafilomycin A_1_ was added to the cells in the last 4 h before harvesting. The graph reports LC3-II levels relative to actin test (I: *n* = 3; CTRL versus no fbs *P = 0.0341 and **P = 0.0026; no fbs versus 24 h IGF-1 **P = 0.0059 and **P = 0.0085). (**J**) HeLa cells were transfected as reported in (A). The western blot panels show the effect of IGF-1R knock-down on the IGF-1/AKT/mTOR pathway. (**K**) HeLa cells were treated for 24 h with MAB391, and then samples were analysed by western blot to check levels of the indicated proteins (see also Supplementary Material, Fig. S1I and J). In all panels, error bars represent standard deviations. (**L** and **M**) HeLa cells seeded on glass coverslips were treated or not with the MAB391 neutralizing antibody. After 24 h, the cells were fixed, permeabilized in methanol, stained with anti-LAMP1 specific antibody and finally analysed by confocal microscope. Two hundred cells for each experimental condition were analysed using a fluorescence microscope, and the number of cells presenting a perinuclear versus peripheral lysosomal distribution was scored. The *P*-value for assessing the variation in lysosomal positioning was determined using Student's *t*-test (*n* = 3; ns, nonsignificant). (**N** and **O**) IGF-1R depletion does not affect acidification of the lysosomal compartment. HeLa cells were seeded on MatTek Petri dish and treated or not with MAB391. After 24 h, the cells were loaded with the LysoSensor as detailed in the Methods section and then analysed by confocal live imaging. The graph shows the mean value with standard deviation of the yellow/blue ratio obtained from 10 confocal fields for each experimental condition, from three independent experiments. Bafilomycin A_1_ was used as a standard positive control (O: *n* = 3; CTRL versus MAB391: ns, nonsignificant; CTRL versus BafA1, ***P = 0.00026).

In order to mimic the potential therapeutic strategies of chronic inhibition of IGF-1 signalling based on the *in vivo* knockout literature (1–4), we initially investigated the effects of knocking down IGF-1R in cell culture systems. Contrary to expectations, IGF-1R knockdown decreased LC3-II levels in HeLa cells grown in a normal medium, as well as in cells treated with saturating levels of bafilomycin A_1,_ which blocks LC3-II degradation, thereby allowing one to infer LC3-II formation rates (11), suggesting that LC3-II/autophagosome formation was reduced (Fig. 1A and B). Furthermore, IGF-1R depletion also decreased autophagosome formation in mouse embryonic fibroblasts (MEFs) derived from IGF-1R hemizygous mice (Supplementary Material, Fig. S1A and B).

The endogenous autophagy substrate p62/sequestosome 1 (14) was increased in cells where IGF-1R was knocked down (Fig. 1C). Interestingly, the accumulation of p62 was autophagy dependent, since this effect was abrogated in the presence of bafilomycin A_1_ (Fig. 1A–C). Another well-known and characterized autophagy substrate is mutant huntingtin with an expanded polyQ tract, associated with Huntington's disease. The percentage of cells with mutant huntingtin aggregates increases linearly as a function of its expression and is increased by autophagy inhibitors (15). Such an effect also occurred upon IGF-1R knockdown (Fig. 1D).

In order to corroborate the effects of IGF-1R inhibition on autophagy, we used a monoclonal antibody (MAB391) that induces time-dependent degradation of the human IGF-1R receptor (16). LC3-II levels in the presence of bafilomycin A_1_ were significantly reduced after 8 h treatment with this antibody and to a greater extent at 24 h (Fig. 1E and F), as were the numbers of GFP-LC3 vesicles (Supplementary Material, Fig. S1C). Note worthily, the reduction in autophagosome formation observed in these experiments is as large as we see when we knockdown key autophagy genes (10). Likewise, MAB391-driven IGF-1R depletion enhanced the accumulation of GFP-Q74 (Supplementary Material, Fig. S1D). Interestingly, we also found that a long-term serum deprivation (8 and 24 h) also reduced autophagosome formation (Supplementary Material, Fig. S1E and F), even though it has been previously described how, under certain metabolic conditions, a short-term serum deprivation can induce autophagy (17,18). This might be compatible with the IGF-1R depletion, since both the strategies would inhibit growth factor-mediated signalling.

We then tested whether medium-term IGF-1R stimulation would modulate LC3-II accumulation in the presence of bafilomycin A_1_ (11), when IGF-1 was added back to cells that had been serum starved for the previous 24 h (since serum starvation depletes cells of growth factors). Importantly, this strategy enables kinetic experiments that are not possible with the knockdown and antibody approaches we used above. While 8 h of IGF-1 supplementation had no clear effects on autophagy (Fig. 1G–I), prolonged (24 h) IGF-1 stimulation resulted in a dramatic increase in LC3-II formation in serum-starved cells, much above the levels observed in serum-starved, non-IGF-1-treated cells (Fig. 1H and I). This effect was compatible with the decreased autophagy we observed upon long-term IGF-1R inhibition (Supplementary Material, Fig. S1E and F). Moreover, we also reproduced this phenomenon in mouse primary cortical neurons (Supplementary Material, Fig. S1G and H).

### Signalling consequences of IGF-1R depletion

We expected IGF-1R inhibition to reduce downstream mTOR activity. mTOR activity resides in two kinase complexes, the mTORC1 and mTORC2. The mTORC1 complex that negatively regulates autophagy also phosphorylates protein p70S6Kinase, and its phosphorylation is widely used to infer mTORC1 activity (19). Surprisingly, IGF-1R siRNA resulted in no obvious effects on either AKT phosphorylation at T_308_, which correlates with the downstream mTOR activation (20), FOXO-3A phosphorylation, which has been reported to transcriptionally modulate autophagy (21), or the phosphorylation of p70S6 K, a direct substrate of the mTORC1 complex (Fig. 1J and Supplementary Material, Fig. S1I). As we observed with IGF-1R siRNA, MAB391 did not decrease p70S6 K phosphorylation, and had no discernible effect on AKT T_308_ phosphorylation (Fig. 1K and Supplementary Material, Fig. S1J). In view of the intimate connection between lysosomes and mTOR signalling (17,22), we also assessed whether lysosomal positioning might be altered in IGF-1R depleted cells, but without observing any significant variation in the intracellular localization of lysosomes (Fig. 1L and M). Furthermore, IGF-1R inhibition did not influence either lysosomal pH (Fig. 1N and O) or the degradation activity of the lysosomal compartment (Supplementary Material, Fig. S1K and L).

### Sustained IGF-1 signalling rescues autophagy inhibition caused by long-term serum deprivation

**Figure 2.**
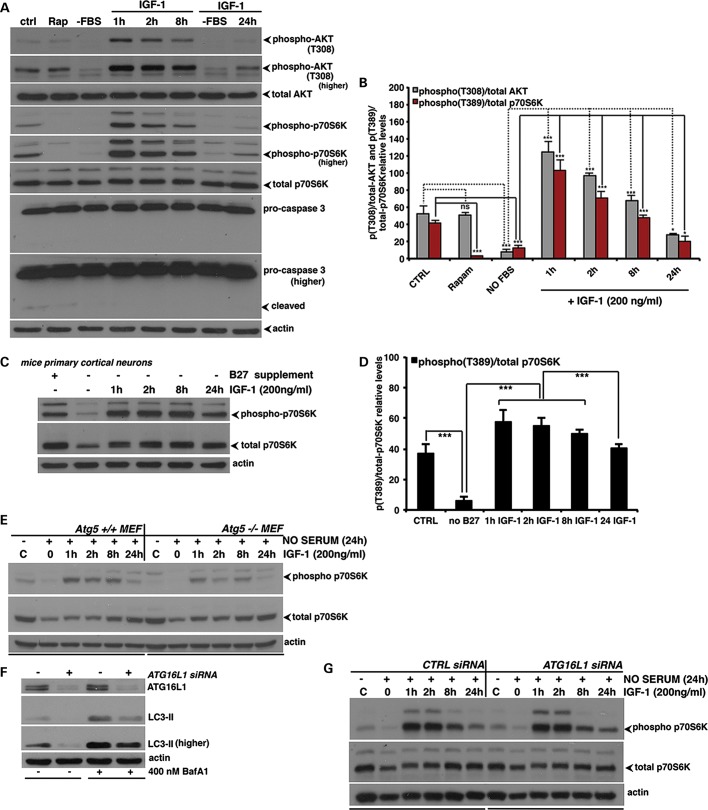
Sustained IGF-1 signalling rescues autophagy inhibition by long-term serum deprivation. (**A** and **B**) HeLa cells were serum starved for 24 h and then stimulated by 200 ng/ml of the IGF-1 R_3_ analogue for the indicated time points. Cells treated with rapamycin (200 nm for 24 h) were included as a positive control for the mTOR inhibition. The western blot panels reporting the effect of serum deprivation and IGF-1 add-back protocol on phospho/total AKT, phospho/total p70S6Kinase levels and caspase-3 cleavage are representative of at least three independent experiments performed in triplicate. The *P*-values of the densitometric analysis were determined by Factorial Anova (*n* = 3; *P < 0.05; ***P < 0.001; ns = nonsignificant). Please note that the serum deprivation (or B27, in the case of primary neuronal cultures) approach we have used in the experiments described in this figure as well as in Supplementary Material, Figure S2, should not be considered as a standard starvation protocol (such as incubation with HBSS or EBSS medium), as the media contains l-glutamine, which would promote autophagy. (**C** and **D**) Mouse primary cortical neurons were deprived of B27 supplement for 24 h and then stimulated by 200 ng/ml of IGF-1 R_3_ for the indicated time points. The western blot panels reporting the effect of B27 deprivation/IGF-1 add-back on phospho/total p70S6 K relative levels are representative of three independent experiments performed in triplicate. The *P*-values of the densitometric analysis were determined by Factorial Anova (*n* = 3; ***P < 0.001). (**E**) ATG5^+/+^ and ATG5^−/−^ MEF cells were serum starved for 24 h and then stimulated by IGF-1 R_3_ for the indicated time points. The western blot panels are representative of at least three independent experiments. (**F**) HeLa cells were transfected for 96 h with either control or anti-ATG16L1 siRNA. The western blot panels report the effect of ATG16L1 knockdown on LC3-II levels. (**G**) Control- or Atg16L1-siRNA transfected HeLa cells were serum starved for 24 h and then stimulated by IGF-1 R_3_ for the indicated time points. The western blot panels reporting the effect of serum deprivation/IGF-1 add-back protocol on the phospho/total p70S6Kinase levels are representative of three independent experiments. In all the panels, error bars represent standard deviations.

The effects of IGF-1R signalling inhibition over 24 h (described in Fig. 1J and K and Supplementary Material, Fig. S1I and J) are different from what we expected to observe in short-term experiments. This suggested a feedback loop, which we investigated by adding back IGF-1 to cells that had been serum starved for 24 h. Serum starvation decreased p70S6 K phosphorylation almost as severely as the mTORC1-specific inhibitor rapamycin (Fig. 2A and B; compare Ctrl versus Rap and Ctrl versus -FBS). However, when we added back IGF-1 to these serum-deprived cells, we observed a surprising result. While, as we would have expected, IGF-1 treatment for 60–120 min resulted in a prompt rise in the phosphorylation of both AKT and p70S6 K, both AKT and mTOR activity (as determined by p70S6 K phosphorylation) decreased dramatically after 24 h of IGF-1 treatment (Fig. 2A and B; please, compare FBS versus 1–8 h and FBS versus 24 h). However, there appeared to be slightly more mTOR activity and AKT phosphorylation in cells treated with IGF-1 for 24 h after 24 h starvation, compared with starved cells (Fig. 2A and B). These effects were not associated with caspase-3 activation (Fig. 2A).

Again, similar phenomena were observed in primary cortical neurons (Fig. 2C and D). In particular, when we removed the B27 supplement (which contains insulin) for 24 h, we observed reduced p70S6 K phosphorylation (Fig. 2C and D), such as in HeLa cells (Fig. 2A and B). When we added back IGF-1 to B27-depleted neurons, we saw a dramatic increase in mTOR activity up to 8 h, which then declined after 24 h (Fig. 2C and D), again mimicking the experiment performed in HeLa cells (Fig. 2A and B).

Importantly, under our experimental conditions these feedback effects of the mTOR activity were largely autophagy independent (23). The increases and then decreases in p70S6 K phosphorylation were observed in serum-starved wild-type and Atg5 null, autophagy-deficient MEFs exposed to IGF-1 for different periods, even though the initial increase in phosphorylation appeared slightly blunted in the Atg5 null MEFs (Fig. 2E), as well as in HeLa cells exposed to either control or ATG16L1 siRNA, which also knocks down a key autophagy gene (Fig. 2F and G). Therefore, the effects of IGF-1R inhibition on downstream signalling were unexpected, but could be explained by compensatory feedback loops involving some critical downstream effectors (IRS-1/-2 and AKT) (please, for further explanations and discussion refer to Supplementary results, Fig. S2 and related references contained in the Supplementary Material section).

### IGF-1R inhibition decreases autophagosome formation by reducing endocytosis

**Figure 3.**
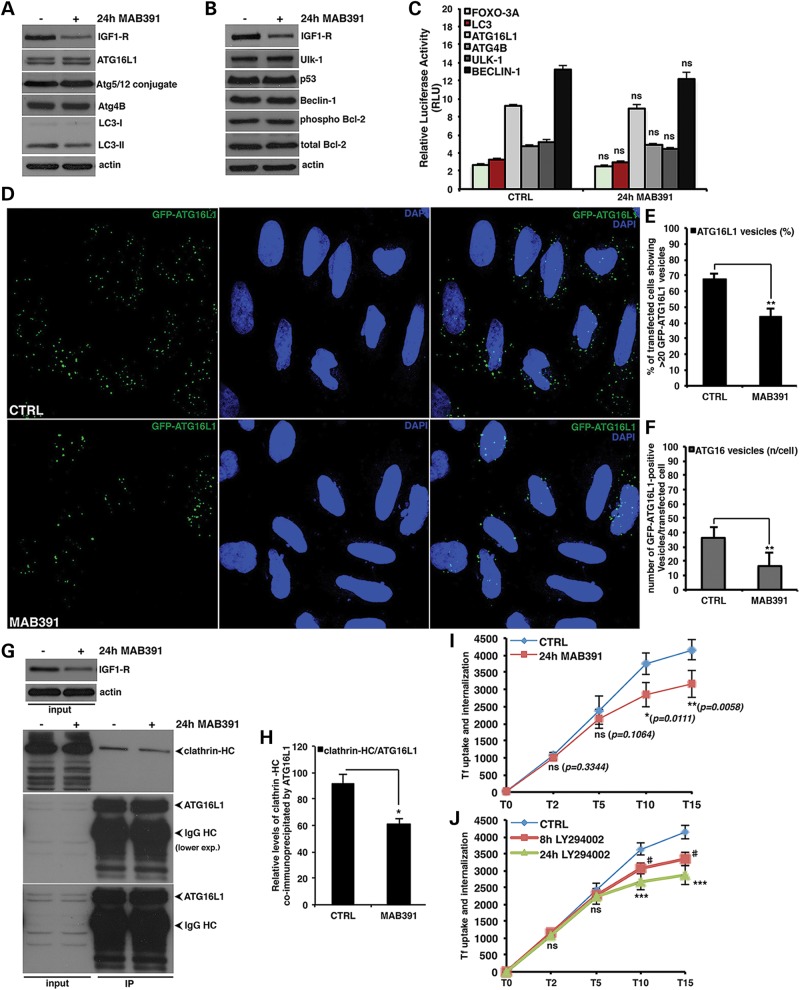
IGF1-R inhibition decreases autophagosome precursor formation by reducing clathrin-dependent endocytosis. (**A** and **B**) HeLa cells were treated for 24 h with the MAB391 antibody to evaluate the effect of the IGF-1R depletion on the levels of the indicated autophagy-related proteins. The western blots are representative of experiments performed in triplicate. (**C**) HeLa cells were transfected with 0.5 μg firefly luciferase reporter plasmids containing the FOXO-3A responsive element or the indicated Atg gene promoters plus 0.05 μg of the Renilla luciferase reporter control plasmid. After 24 h, the cells were treated for a further 24 h with MAB391. RLU values are reported as the average and standard deviations of at least three independent experiments carried out in triplicate. Statistical analysis was performed using Student's *t* test (*n* = 3; CTRL versus 24 h MAB391, FOXO-3A: *P* = 0.2825, LC3: *P* = 0.1707, ATG16L1: *P* = 0.2268, ATG4B: *P* = 0.0672, ULK-1: *P* = 0.0768, BECLIN-1: *P* = 0.1769). (**D–F**) HeLa cells were transfected with 0.5 μg of the GFP-Atg16L1 construct. After 24 h, the cells were treated for 24 h with MAB391, fixed and analysed under fluorescence microscope. The *P*-values for assessing the percentage of cells showing ≥20 GFP-Atg16-positive vesicles as well as the number of GFP-Atg16 vesicles/cell were determined by Student's *t*-test (E: *n* = 3; CTRL versus 24 h MAB391, *P* = 0.00272; F: *n* = 3; CTRL versus 24 h MAB391, *P* = 0.00496). (**G** and **H**) HeLa cells were treated for 24 h with MAB391 and then lysed as detailed in the Methods section. Equal amounts of total protein were immunoprecipitated with the anti-ATG16L1 antibody and the amount of interacting clathrin heavy chains assessed by western blot analysis. The graph in (H) shows the quantitative analysis of endogenous clathrin heavy chain co-immunoprecipitated by Atg16L1. The *P*-value of the densitometric analysis was determined by Student's *t*-test on three independent experiments (G: *n* = 3; CTRL versus 24 h MAB391, *P* = 0.0223). (**I** and **J**) HeLa cells were treated for 24 h with 10 μg/ml MAB391 or LY294002 (10 μM) for the indicated times. The binding and internalization assays for the fluorochrome-conjugated human transferrin were then performed as described in the Methods section. The amount of internalized ligand was measured by FACS analysis. The graphs report the total amount of internalized transferrin (Tf) at different time points (as indicated), and normalized to the amount of Tf bound to the cognate receptor at the cell surface at Time 0 (i.e. before the temperature shift). The *P*-values to assess the differences in the rate of internalization of transferrin receptor were determined by Student's *t*-test on five independent experiments (I: *n* = 5, *P*-values are shown in the figure; J: *n* = 5; CTRL versus 8 h LY294002, T10, *P* = 0.0012; T15, *P* = 0.0017; CTRL versus 24 h LY294002, T10, *P* = 0.00022, T15, *P* = 0.00031). In all the panels, error bars represent standard deviations.

Since the signalling data did not provide a simple explanation for the unexpected effects of IGF-1R inhibition on autophagy, we considered alternative mechanisms. Despite consistently reducing LC3-II levels (Fig. 3A) and the numbers of LC3 vesicles (Supplementary Material, Fig. S3A–C), MAB391 did not affect the levels of the autophagy-related proteins Atg16L1, Atg4B, Beclin-1, Ulk-1 (6), the levels of the Atg5-12 conjugate that regulate LC3-II formation (24), or the levels of phosphorylated Bcl-2 (25), and p53 that regulate autophagy (26) (Fig. 3A and B). In addition, we observed no change either in transcriptional activation of the FOXO-3A responsive element or in the LC3, Atg16L1, Atg4B, Ulk-1 and Beclin-1 promoters upon IGF-1R depletion (Fig. 3C).

Interestingly, although IGF-1R depletion did not alter Atg16L1 protein levels (Fig. 3A), it decreased the numbers of GFP-Atg16L1-positive vesicles in cells after MAB391-mediated depletion of the IGF-1R receptor (Fig. 3D–F). We used GFP-Atg16L1 here, since one cannot detect quantifiable numbers of these vesicles using antibodies to endogenous protein under basal conditions; while this can be done by inducing autophagy using full amino acid starvation, this perturbation would not be feasible for an experiment aiming to test the effects of IGF-1R depletion. However, we have extensively validated the use of this approach (9,10). Interestingly, the interaction between clathrin heavy chain and endogenous Atg16L1 protein was reduced in cells depleted of the IGF1-R receptor (Fig. 3G and H). Thus, the lower amount of LC3-II (i.e. mature autophagosomes) present in IGF-1R depleted cells can be, at least in part, explained by a reduced formation of autophagosomal precursors at the plasma membrane. We tested whether this phenotype was associated with a concomitant reduction in the rate of endocytosis (27). Compared to control, cells treated with the MAB391 antibody had significantly decreased transferrin receptor internalization (Fig. 3I), an assay used to measure the dynamics of clathrin-dependent endocytosis (28). Likewise, we showed a time-dependent reduction in the rate of transferrin receptor internalization in cells treated with the PI3-kinase inhibitor LY-294002, suggesting that the observed effect might be dependent on the activity of the Class I PI(3)K (Fig. 3J). We then asked whether IGF-1R agonism might have an effect on endocytosis. Surprisingly, and consistent with the effect of short- and long-term IGF-1 stimulation on autophagy (Fig. 2 and Supplementary Material, Fig. S2), we observed reduced endocytosis in serum-starved cells (Supplementary Material, Fig. S3D; compare CTRL versus NO FBS). Moreover, a prolonged (24 h) but not short-term (2 h) IGF-1 stimulation of previously serum-starved cells enabled recovery of endocytosis (Supplementary Material, Fig. S3D; compare NO FBS with 2 and 24 h IGF-1), consistent with this paradigm being able to recover (upon long-term stimulation) the autophagosome formation (see also Fig. 1G–I and Supplementary Material, Fig. S1G and H). Thus, IGF-1R depletion inhibits endocytosis and the interaction of Atg16L1 with clathrin, which would, in turn, decrease early autophagosome precursor formation at the plasma membrane (9).

### IGF-1R modulates mTORC2-PKCα/β -actin cascade to regulate autophagy

**Figure 4.**
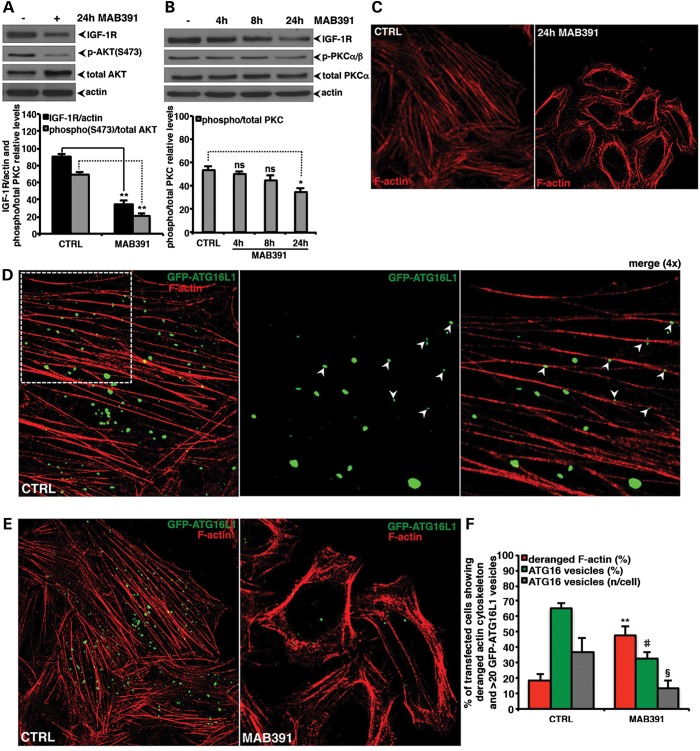
IGF-1R depletion reduces Atg16L1 vesicles formation by impairing the actin cytoskeleton dynamics. (**A** and **B**) HeLa cells were treated for the indicated time points with MAB391. Samples were then assayed for evaluating the effect of IGF-1R depletion on the levels of the indicated proteins. The western blots are representative of experiments performed in triplicate. The graphs in (A) and (B) report the IGF-1R to actin, phospho(S473)/total AKT and phospho/total PKCα relative levels, respectively. The *P*-values were determined by two-tailed Student's *t*-test (A: *n* = 3; CTRL versus 24 h MAB391; IGF-1R **P = 0.00378; P-AKT **P = 0.00586) (B: p-PKCα *n* = 3; CTRL versus 4 h MAB391 *P* = 0.43301; CTRL versus 8 h MAB391 *P* = 0.14323; CTRL versus 2 h MAB391 *P = 0.02914). (**C**) HeLa cells were treated or not with MAB391. After 24 h, the cells were fixed, permeabilized, stained with phalloidin and finally analysed by a confocal microscope. (**D–F**) HeLa cells seeded on glass coverslips were transfected with the 0.5 μg of GFP-ATG16 vector and treated or not with 10 μg/ml of the MAB391 neutralizing antibody. After 24 h, the cells were fixed, permeabilized, stained with phalloidin and finally analysed by a confocal microscope. The *P*-values for assessing the number of GFP-ATG16 vesicles, the percentage of cells showing ≥20 GFP-Atg16 vesicles and the actin cytoskeleton derangement were determined by Student's *t*-test (F: *n* = 3, CTRL versus 24 h MAB391, actin derangement: **P = 0.00244; percentage of cells with ≥20 ATG16 vesicles/cell: ^#^*P* = 0.00228; number of GFP-ATG16 vesicles/cell: ^§^*P* = 0.00336). In all the panels, error bars represent standard deviations.

**Figure 5.**
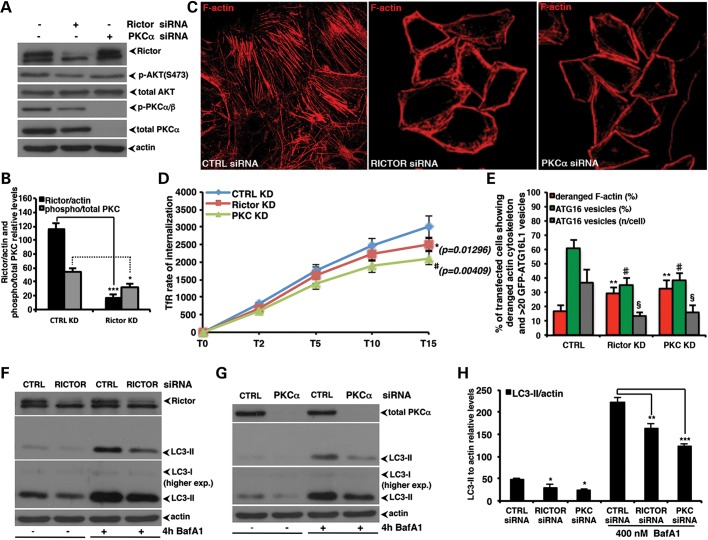
The Rictor/PKCα cascade controls the autophagosome formation by regulating the rate of endocytosis. (**A** and **B**) HeLa cells were transfected with control, Rictor, or PKCα siRNA. After 96 h, the cells were lysed and the samples subjected to western blot analysis to evaluate the effect of knockdown on the indicated proteins. The western blots are representative of experiments performed in triplicate. The graph in (B) reports the rictor/actin, and phospho/total PKCα relative levels, respectively. The *P*-values were determined by two-tailed Student's *t*-test (B: *n* = 3; CTRL versus Rictor kd; Rictor ***P = 0.00089; p-PKC **P = 0.01225) (**C**) HeLa cells seeded on glass coverslips were transfected as indicated in A. After 96, cells were fixed, permeabilized, stained with phalloidin and finally analysed by a confocal microscope. (**D**) HeLa cells were transfected with control, rictor, or PKCα siRNA. After 96 h, the binding and internalization assays of the fluorochrome-conjugated human transferrin were then performed. The *P*-values to assess differences in the internalization of transferrin receptor were determined by Student's *t*-test on six independent experiments (*n* = 6, *P*-values relative to T15 are shown in the figure). (**E**) The *P*-values for assessing the number of GFP-Atg16 vesicles, the percentage of cells showing ≥20 GFP-Atg16 vesicles and the actin cytoskeleton derangement were determined by Student's *t*-test (*n* = 3; actin derangement: Rictor kd, *P* = 0.0015, PKCα kd, *P* = 0.0049; percentage of cells with ≥20 ATG16 vesicles: rictor kd, *P* = 0.0136, PKCα kd, *P* = 0.0231; number of ATG16 vesicles: Rictor kd, *P* = 0.00122, PKCα kd, *P* = 0.00421). (**F–H**) HeLa cells were transfected for 96 h with 50 nm of control, rictor or PKCα siRNA. Bafilomycin A_1_ was added to the cells in the last 4 h before harvesting. The graph shows the LC3-II levels relative to actin panels, error bars represent standard deviations (*n* = 3; Ctrl versus Rictor kd: *P* = 0.0279 and *P* = 0.0079 in the presence of BafA_1_; Ctrl versus PKCα kd: *P* = 0.0145 and *P* = 0.0005, respectively).

In order to understand how IGF-1R signalling could modulate autophagy via endocytosis, we considered the mTORC2 pathway, a known downstream effector of IGF-1R, which has not been linked clearly with autophagy (please, see supporting note and related references). As expected, IGF-1R depletion decreased the activity of this complex, as it reduced the downstream phosphorylation at the mTORC2-dependent site (serine 473) on AKT (Fig. 4A). The mTORC2 complex is known to regulate actin dynamics via protein kinase C α/β (PKCα/β) (29–31) and MAB391 treatment resulted in decreased PKCα/β activity (Fig. 4B), as well as obviously altered actin cytoskeleton (Fig. 4C). Notably, we observed a striking correlation between actin cytoskeleton morphology and the number of Atg16L1-positive vesicles (Fig. 4D). In particular, the actin cytoskeleton derangement consequent to MAB391 treatment was associated with decreased numbers of Atg16L1 autophagosome precursors (Fig. 4E and F). By treating cells with the actin polymerization inhibitor latrunculin A, we confirmed that the actin cytoskeleton is required for effective endocytosis (32) (Supplementary Material, Fig. S4A) and, consistent with recent observations (33), showed that it is involved in early events of autophagosome formation (Fig. S4B–E). We therefore tested this potential mechanism in greater depth. Genetic knockdown of Rictor, a critical component of the rapamycin-insensitive mTORC2 (but not mTORC1) complex, reduced PKCα/β \xEF\x80phosphorylation (Fig. 5A and B), and both Rictor and PKCα knockdown severely disrupted the actin cytoskeleton (Fig. 5C), reduced endocytosis (Fig. 5D), lowered the numbers of Atg16L1 vesicles (Fig. 5E) and, as a consequence, decreased autophagosome formation (Fig. 5F–H). Notably, Rictor selective knockdown had no effects on mTORC1 signalling (Supplementary Material, Fig. S5A and B). Moreover, long-term treatment (8–24 h) with Torin-1, a novel mTORC1 and mTORC2 catalytic inhibitor (34), decreased autophagosome formation, while inhibiting (differently from MAB391) mTORC1 activity (Supplementary Material, Fig. S5C–E). All together, these data show how mTORC2 and PKCα/β  inhibition, and disruption of the actin cytoskeleton are sufficient to impair autophagosome precursor biogenesis, and thus can account, at least in part, for the autophagy-inhibitory effects of reducing IGF-1R signalling.

### IGF-1R inhibition attenuates autophagy *in vivo*

**Figure 6.**
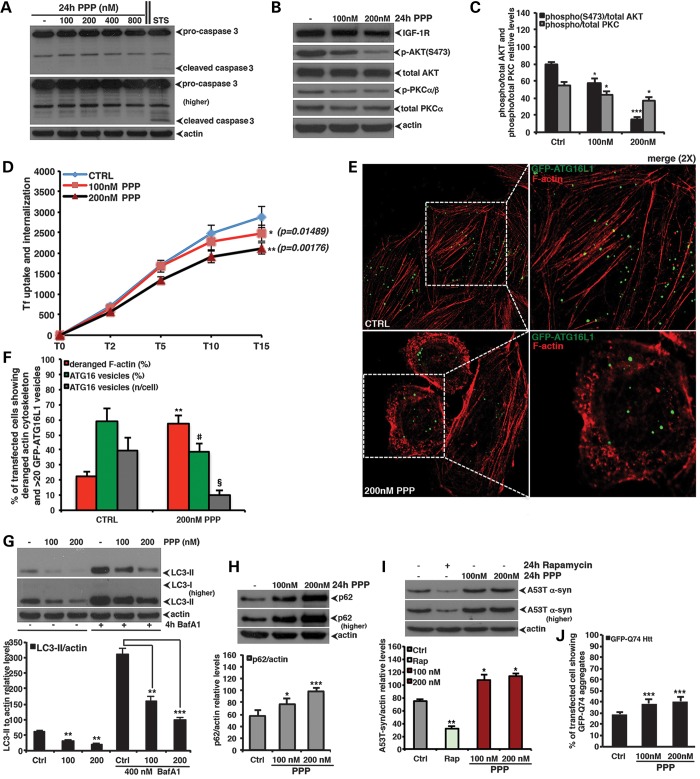
Chemical inhibition of IGF-1R signalling inhibits autophagy. (**A**) PPP toxicity assessment. The orally active IGF-1R selective inhibitor PPP was dissolved in dimethyl sulfoxide (DMSO) and added to normally growing HeLa cells for 24 h at the indicated dosages. Cells were then lysed and analysed by western blot for the caspase-3 cleavage. In these experiments, staurosporine (8 h at 1 μm) was included as a positive control for the activation of the apoptotic cascade. (**B** and **C**) HeLa cells were treated for 24 h with the indicated concentrations of the selective IGF-1R inhibitor PPP. Samples were then subjected to western blot analysis to evaluate the effect of PPP on the indicated proteins. The western blots are representative of experiments performed in triplicate. The graph in (C) reports the phospho(S473)/total AKT and phospho/total PKCα relative levels, respectively. The *P*-values were determined by a two-tailed Student's *t*-test (*n* = 3; CTRL versus 100 nm PPP, p-AKT *P = 0.03099; p-PKCα *P = 0.04724; CTRL versus 200 nm PPP p-AKT ***P = 0.00019; p-PKCα *P = 0.02037). (**D**) HeLa cells were treated for 24 h with the indicated concentrations of PPP and endocytosis of transferrin receptor analysed. The *P*-values to assess differences in the internalization of transferrin receptor were determined by Student's *t*-test (*n* = 5, *P*-values relative for transferrin uptake at T15 in PPP-treated, compared with control cells, are shown in the main figure). (**E** and **F**) HeLa cells transfected with the GFP-ATG16 vector were treated or not with 200 nm of PPP. After 24 h, the cells were fixed, permeabilized in 0.1% Triton X-100, stained with phalloidin and finally analysed by confocal microscopy. The *P*-values for assessing the number of GFP-ATG16 vesicles, the percentage of cells showing ≥20 GFP-Atg16 vesicles and the actin cytoskeleton derangement were determined using Student's *t*-test (*n* = 3; actin derangement: Ctrl versus PPP, *P* = 0.0013; percentage of cells showing ≥20 GFP-Atg16 vesicles: Ctrl versus PPP, *P* = 0.0121; number of GFP-ATG16 vesicles/cell: §*P* = 0.00629) (**G**) HeLa cells were treated for 24 h with the indicated concentrations of PPP. Bafilomycin A_1_ was added to the cells in the last 4 h before harvesting. The graph shows the LC3-II levels relative to actin (*n* = 3; 100 nm PPP: *P* = 0.0081; 200 nm PPP: *P* = 0.0062, in the absence of BafA_1_; 100 nm PPP: *P* = 0.0031; 200 nm PPP: *P* = 0.0005, in the presence of BafA1). In all the panels, error bars represent standard deviations. (**H**) HeLa cells were treated as already described in (B). The graph in (I) reports the p62 levels relative to actin. The *P*-values were determined by two-tailed Student's *t*-test (*n* = 3; CTRL versus 100 nm PPP; *P = 0.0279; ***P = 0.00086). (**I**) PPP impairs clearance of mutant (A53 T) α-synuclein in stable inducible PC12 cells. The A53 T α-synuclein transgene was induced with doxycycline for 48 h and then switched off (by antibiotic removal) before the cells were treated with the vehicle alone (DMSO), rapamycin (200 nm) or PPP at the indicated concentrations for a further 24 h. The graph in (I) reports the A53T-synuclein levels relative to actin. The *P*-values were determined by a two-tailed Student's *t*-test (*n* = 3; CTRL versus rapamycin **P = 0.0087; CTRL versus 100 nm PPP *P = 0.0469; CTRL versus 200 nm PPP *P = 0.0122). (**J**) HeLa cells were transfected with 2 μg of the GFP-HD74 vector for 48 h. In the last 24 h, the cells were treated or not with the indicated concentrations of PPP. The *P*-value for assessing the EGFP-HDQ74 aggregation was determined using Student's *t*-test (*n* = 3; CTRL versus 100 nm PPP, ***P = 0.00073; CTRL versus 200 nm PPP, ***P = 0.00019).

In order to assess whether our cell-based data were relevant *in vivo*, where one might envisage IGF-1R inhibition to be employed as a therapeutic strategy, we studied the effect of picropodophyllin (PPP), a selective IGF-1R inhibitor (Supplementary Material, Fig. S6) (35,36). Among the various IGF-1R chemical inhibitors that are currently available, we decided to focus on the cyclolignans family member PPP, because of previous reports showing specific inhibition of IGF-1R tyrosine phosphorylation and activity, without effects on other relevant tyrosine kinase receptors (35). In particular, cyclolignans can specifically and efficiently reduce the auto-phosphorylation, activity and hence downstream signalling of IGF-1R in different cell lines; furthermore, PPP has been shown to be virtually non-toxic in animal models (35,36). Consistent with IGF-1R knockdown and MAB391 treatment, non-toxic doses of PPP (Fig. 6A and Supplementary Material, Fig. S6) attenuated mTORC2 activity (i.e. AKT-S_473_ phosphorylation) and the PKCα/β phosphorylation (Fig. 6B and C), perturbed the actin cytoskeleton morphology, reduced endocytosis and Atg16L1 vesicle numbers (Fig. 6D–F) and, as a consequence, inhibited autophagosome formation (Fig. 6G). The slight decrease of total PKCα inn PPP-treated cells might be due to a concomitant enhanced degradation (37,38). Consistent with these data, PPP treatment increased the intracellular levels of selective autophagy substrates, such as p62 protein (Fig. 6H) and mutant A53 T α-synuclein (Fig. 6I), as well as the proportion of cells with GFP-Q74 mutant huntingtin aggregates (Fig. 6J). Interestingly, since PPP affects IGF-1R activity (i.e. phosphorylation of downstream modules) but not its own levels (Fig. 6B), such experimental approach allow us to dissociate any effect observed upon inhibition of IGF-1R signalling from those that might rather depend on the IGF-1R receptor degradation. Nonetheless, the PPP data are entirely consistent with what we have reported above with either genetic knockdown of the receptor or MAB391 treatments in terms of modulation of mTORC2-related signalling and autophagy.

**Figure 7.**
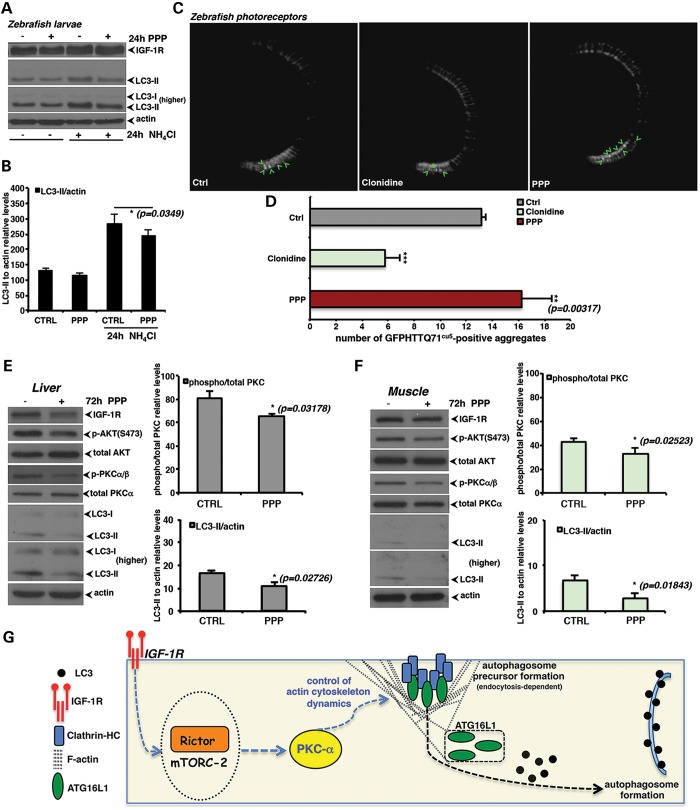
IGF-1R signalling perturbation reduces autophagy *in vivo*. (**A** and **B**) PPP inhibits autophagic flux in zebrafish larvae. Ammonium chloride (NH_4_Cl) causes significant increases in levels of LC3-II in zebrafish larvae, consistent with an ability to block autophagosome/lysosome fusion. Co-treatment with PPP significantly decreases the levels of LC3-II in the presence of ammonium chloride. The panel shows western blots for IGF-1R and LC3-II against actin, used as loading control. The graphs represent densitometric analyses of three independent experiments (*n* = 3; *P* = 0.0349). (**C** and **D**) Aggregate counting was performed using the heterozygous larvae from the transgenic (rho:EGFP-HTT71Q)^cu5^ zebrafish line. From 3 d.p.f. to 7 d.p.f., transgenic HD zebrafish larvae were dark-reared in embryo medium alone or embryo medium containing either DMSO, 30 μm clonidine or 100 µm PPP. At 7 d.p.f., larvae were anaesthetized and fixed in 4% paraformaldehyde (PFA). Larvae were washed, embedded in OCT medium and frozen on dry ice for subsequent cryosectioning. The total number of GFP-positive aggregates was counted over 100 μm of the central retina, either side of the optic nerve. Sections were viewed and representative images acquired using a GX Optical LED fluorescent microscope. Numbers of positive aggregates were scored and mean values were calculated for each treatment group (*n* = 4 fish/8 eyes; DMSO versus PPP: *P* = 0.00317)*.* (**E** and **F**) PPP inhibits autophagy and reduces steady-state levels of LC3-II in mice. Mice were subjected to intra-peritoneal injection with either PPP (20 mg/kg/24 h) for 3 days. Following sacrifice, the liver and muscle were homogenized and samples analysed by western blotting for levels of the indicated proteins. The graphs represent the mean results of the phospho/total PKCα and LC3-II levels relative to actin from three mice per group (E: liver, *n* = 3; p-PKCα  *P* = 0.03178, LC3-II *P* = 0.02726) (F: muscle, *n* = 3; p-PKCα  *P* = 0.02523, LC3-II *P* = 0.01843). In all the panels, error bars represent standard deviations. (**G**) Proposed model for the IGF-1R signalling-dependent modulation of the autophagic pathway. IGF-1R depletion reduces autophagy and has no effect on the canonical mTORC1 complex-mediated signalling pathway. The reduced amount of autophagosomes present in IGF-1R depleted cells can be, at least in part, explained by a reduced formation of autophagosomal precursors at the plasma membrane. In particular, IGF-1R depletion inhibits mTORC2, which in turn reduces the activity of PKCα/β. This perturbs the actin cytoskeleton dynamics and decreases the rate of clathrin-dependent endocytosis, which negatively impacts autophagosome precursor formation. This has important consequences for the interpretation of genetic experiments in mammalian systems and for evaluating the potential of modulating the signalling through this pathway for therapeutic purposes.

We initially assessed the effects of PPP *in vivo* in a zebrafish model, where we have previously optimised the use of NH_4_Cl as an autophagosome degradation blocker analogous to bafilomycin A_1_ (39). PPP decreased autophagosome formation in zebrafish larvae (Fig. 7A and B). Consistent with these data, PPP treatment increased the number of aggregates of mutant huntingtin expressed in the zebrafish rod photoreceptors (Fig. 7C and D), in a manner similar to what we previously observed with other autophagy inhibitors (39,40). This also contrasts with the reduction in aggregate numbers in this model seen with autophagy inducers, as we have observed previously (41), and as shown here with clonidine (Fig. 7C and D). In mice, intra-peritoneal administration of PPP effectively decreased AKT phosphorylation (S_473_) as well as PKCα/β phosphorylation, and reduced LC3-II levels in mouse liver (Fig. 7E) and muscle (Fig. 7F). Overall, the effects exerted by PPP *in vivo* appear to be biologically significant—for instance, in the zebrafish the huntingtin aggregate counts increased by 30% (Fig. 7D) whereas in the mouse muscle, there appeared to be a reduction of LC3 by ∼50% (Fig. 7F). Hence, IGF-1R antagonism can effectively down modulate the activity of the autophagic pathway *in vivo*.

## DISCUSSION

The IGF-1R pathway has received extensive attention as its inhibition increases lifespan and ameliorates neurodegenerative diseases in a range of model organisms (1–4). Furthermore, the potential druggability of the receptor suggests that it may be suitable for therapeutic targeting. Nonetheless, our data suggest that serious caution is warranted with this approach. IGF-1R inhibition decreases mTORC2, which, in turn, reduces the activity of PKCα/β. This perturbs the actin cytoskeleton and decreases the rate of endocytosis, which impacts autophagosome precursor formation (summarized in Fig. 7G). Thus, contrary to expectations, IGF-1R inhibition has liabilities with regard to autophagy. These data are consistent with a study from Yamamoto *et al*., who showed that IRS2 positively regulates mutant huntingtin clearance in an autophagy-dependent manner, although this study did not make connections with IGF-1R or mTORC2 signalling (42). Our findings have important consequences for the interpretation of genetic experiments in mammalian systems and for evaluating the potential of targeting the receptor and/or modulating signalling through the downstream pathway for therapeutic purposes. For instance, this additional mechanism would likely have impact on the overall efficacy of IGF-1R down-modulation with respect to neurodegenerative diseases, where autophagy is beneficial (7,13,15,41–43). This may even be relevant in terms of longevity, since IGF-1R signalling inhibition does not appear to have clear effects on lifespan in male mice (44), leading these authors to suggest that the effects of inhibiting this pathway in mammals may not be as straightforward as has been predicted by invertebrate studies (44). One may be able to bypass this additional effect by targeting downstream effectors of the IGF-1R pathway, although these should also be tested for unforeseen side effects. Moreover, from a pharmacological point of view, such modules could result more challenging to be targeted than the receptor itself. Indeed, it is tempting to speculate that one may be able to achieve synergistic benefits by inhibiting the key effectors of the IGF-1R pathway, alongside pharmacological stimulation of the autophagic pathway. Finally, our data also suggest that there may be benefits in using dual mTORC1/2 catalytic inhibitors if administered over longer periods, as these may result in inhibiting autophagy, which may decrease viability of at least some types of cancers (43,45,46).

## MATERIALS AND METHODS

### Antibodies

The following antibodies have been used in this work: anti-LC3 (Novus Biological); anti-human IGF-1R neutralizing antibody (MAB391, R&D Systems); mouse monoclonal anti-LC3 (Nanotools); mouse monoclonal anti-LAMP1 (clone H4A3, obtained from Developmental Studies Hybridoma Bank, University of Iowa); anti-IGF-1R, anti-IRS-1, anti-IRS-2, anti-bcl2, anti-p53, anti-phospho(T308)-AKT (SantaCruz) anti-Beclin-1, anti-phospho-Bcl2, anti-Ulk1, anti-caspase-3, anti-phospho(T389) and total-p70S6kinase, anti-phospho(S473) and total-AKT, anti-phospho-PKC(α/β) and total-PKCα; anti-Rictor, anti-phospho- and total-FOXO3A (Cell Signal); anti-human sestrin-1 antibody (Abnova); anti-clathrin heavy chain and anti-p62 (BD Bioscience); anti-Atg16L1 (MBL); anti-Atg5 and anti-actin (Sigma); anti-Atg4B (AbCam); anti-HA antibody (Covance); anti-mouse and anti-rabbit HRP-conjugated secondary antibodies (GE Healthcare), AlexaFluor594- and AlexaFluor488-conjugated goat anti-mouse; AlexaFluor488-conjugated goat anti-rabbit secondary antibodies, AlexaFluor647-conjugated human transferrin and AlexaFluor594-conjugated phalloidin were from Molecular Probes (Invitrogen).

### Constructs

The EGFP-HDQ74 vector was characterized previously (47); the EGFP-LC3 and EGFP-ATG16L1 were kind gifts from Yoshimori. The luciferase reporter construct under the control of the synthetic Forkhead promoter (FOXO-3A luciferase) was a kind gift of Brunet (48). The luciferase vector containing the human Beclin-1 promoter region has been described elsewhere (49). The human LC3, Atg16L1, Atg4B and Ulk-1 promoter vectors were obtained from Yoshida (50). The Renilla luciferase vector was purchased from Promega. The chimeric immunoglobulin receptor expression vector (Fcγ-RI-γ) has been described previously (51).

### Chemicals

The human IGF-1R, Rictor, PKCα and ATG16L1 *SMART* pool siRNA reagent (Dharmacon) were used at a final concentration of 50nm. The anti-human IGF-1R MAB391 antibody (16) was used at a final concentration of 10 μg/ml. The IGF-1 R_3_ analogue (Sigma) was used at a final concentration of 200 ng/ml. Bafilomycin A_1_ (Millipore) and rapamycin (Sigma) were used at a final concentration of 400 and 200 nm, respectively. Torin 1 (kindly provided by Gray and Sabatini) was dissolved in dimethyl sulfoxide (DMSO) and used at 200 nm. LY294002 and Latrunculin A (Sigma) were used at final concentrations of 10 and 1 μm, respectively. MG132 (Sigma) was used at 10 μm. The IGF-1R selective inhibitor picropodophyllotoxin (PPP, Tocris) was dissolved in DMSO and used at the indicated concentrations. Clonidine (Sigma) was dissolved in DMSO and used at 30 μm. LysoSensor Yellow/Blue DND-160 was from Molecular Probes (Invitrogen).

### Cell culture

HeLa, ATG5^+/+^ and ATG5^–/−^ MEF, IGF-1R^+/+^ and IGF-1R^+/−^ MEF cells were grown at 37°C in 10% FBS, 2 mm l-glutamine, pen/strep supplemented Dulbecco's modified Eagle's medium (DMEM). For the IGF-1 forward signalling experiments, HeLa and MEF cells were serum-deprived for 24 h, and then the IGF-1 R_3_ was added under the same media conditions, for different time points ranging from 1 to 24 h, as detailed in the Results section.

### Isolation and culture of mouse primary cortical neurons

Primary cortical neurons were isolated from C57BL/6 mice embryos at E16.5 (Jackson Laboratories). Briefly, pup brains were harvested and placed in ice-cold DMEM where the meninges were removed and the cerebral cortices were dissected and incubated in DMEM. After mechanical dissociation using sterile micropipette tips, dissociated neurons were resuspended in DMEM and centrifuged. Cell count and viability assay were performed using the trypan blue exclusion test. Viable cells were seeded on poly-D-lysine and laminin-coated 6-multiwell plates (7.5 × 10^5^ cells per well). Cells were cultured in DMEM supplemented with 2 mm glutamine, 2% B27 supplement and 1% Penicillin-Streptomycin-Fungizone (PSF; Invitrogen) at 37°C in a humidified incubator with 5% CO_2_ and 95% O_2_. One half of the culturing medium was changed every two days until treatment. After 7 days of culturing *in vitro*, differentiated cortical neurons were B27-deprived for 24 h and then stimulated with the IGF-1 R_3_ analogue as indicated in the relevant figure legends. For the assessment of autophagy by LC3-II levels, a saturating concentration (400 nm) of bafilomycin A_1_ was added to the cells in the last 4 h before harvesting.

### Transfection

In all the RNA interference experiments, HeLa cells were transfected 24 h after seeding with a 50 nm final concentration of the indicated *SMART*pool siRNAs using Lipofectamine 2000, according to the manufacturer's instructions (Invitrogen). Cells were then cultured in a full medium for 72–96 h. For the assessment of autophagy by LC3-II levels, a saturating concentration (400 nm) of bafilomycin A_1_ was added to the cells in the last 4 h before harvesting (52). For the EGFP-HD74 aggregation experiments upon IGF-1R knockdown, HeLa cells were first transfected as above reported. Forty-four hours after the first round of transfection, the cells were re-transfected with the following combination: EGFP-HDQ74 plus either control or specific siRNA (2 μg: 50 nm) and kept in culture for the next 48 h. To assess the effect of the neutralizing antibody, HeLa cells were transfected with 2 μg of the EGFP-HD74 for 48 h and then challenged or not with MAB391 for 24 h. Cells were finally washed and fixed with 4% paraformaldehyde (Sigma), mounted in ProLong Antifade (Invitrogen) and observed with a fluorescence microscope. For the GFP-LC3 dots experiments, HeLa cells were transfected with 0.5 μg of the EGFP-LC3 for 24 h, challenged or not with MAB391 for the following 24 h and then fixed, mounted on coverslips and analysed with a fluorescence microscope.

### IGF-1R knockdown and depletion experiments

In all the RNA interference experiments, cells were transfected with a 50 nm final concentration of the *SMART* pool siRNAs (Dharmacon) using Lipofectamine 2000 (Invitrogen). For the IGF-1R depletion experiments, HeLa cells were treated at different time points with the anti-human IGF-1R MAB391 at a final concentration of 10 μg/ml.

### Transfection experiments for luciferase reporter assays

HeLa cells were seeded in six multiwells and transfected with 0.5 μg of the indicated luciferase reporter vectors plus 0.05 μg of the renilla luciferase and cultured in a full medium for 24 h. Cells were then treated or not with the MAB391 neutralizing antibody for 24 h and finally lysed in reporter lysis buffer (Promega). Firefly and Renilla luciferase activities were measured in a luminometer using the Dual-Glo luciferase assay kit (Promega). The relative luciferase activity (RLU) is defined as the firefly-to-renilla luciferase activity ratio and normalized for the protein concentration of each sample. In all experiments, the values are reported as the average and standard deviations of at least three independent experiments carried out in triplicate. Statistical analysis was performed using Student's *t*-test.

### Western blot analysis

Cells were washed and harvested in ice-cold PBS and pellets were lysed on ice in Laemmli buffer for 30 min, in the presence of a protease/phosphatase inhibitors mix (Roche). Protein samples were boiled for 5–7 min at 100°C, separated by SDS–PAGE, and then subjected to western blot analysis.

### Co-immunoprecipitation assays

To analyse the ATG16L1/clathrin heavy-chain interaction, HeLa cells were lysed on ice for 30 min in Buffer B (10 mm Tris–HCl, 150 mm NaCl, 1 mm EDTA, pH: 8.0), supplemented with protease and phosphatase inhibitors mix, in the presence of 1% Triton X-100. Lysate were then cleared by centrifugation and equal amounts (1.5 mg) of total protein were incubated for 16 h with the anti-ATG16L1 antibody (1 : 200) on a rotating wheel. Immunocomplexes were isolated with protein G-sepharose (GE-Healthcare Amersham), extensively washed, resuspended and boiled in Laemmli sample buffer, separated by SDS–PAGE and then subjected to western blot analysis.

### Analysis of autophagy

Assessment of autophagic flux by endogenous LC3-II levels or vesicles, eGFP-LC3 or eGFP-ATG16L1 vesicles and the clearance of exogenous autophagy substrates (A53 T α-synuclein and GFP-HDQ74 exon 1 huntingtin) were performed as detailed below.

### Quantification of poly-Q74 huntingtin aggregates formation, LC3-positive and Atg16L1-positive vesicles

Quantification of aggregate formation and LC3 dots were assessed as already previously described (16,52). Two hundred EGFP-HDQ74 transfected cells were selected and the number of cells with aggregates counted using a fluorescence microscope. For quantification of LC3-II dots upon transfection, 200 EGFP-LC3 positive HeLa cells were selected and cells with ∼20 or more LC3-labelled vesicles were counted. Quantification of cells showing GFP-Atg16L1 vesicles was performed as previously described (9,10). Briefly, the number of GFP-ATG16L1 per transfected cell was scored, and then the percentage of cells showing at ≥20 GFP-ATG16L1-positive vesicles was also calculated and plotted. The identity of the slides was unavailable to the observer until all slides had been studied. The experiments were performed in triplicate and repeated at least three times. Quantification of endogenous LC3-II dots was performed as previously described (53). Two hundred cells for each experimental condition were analysed by fluorescence microscopy and number of endogenous LC3 dots were scored in a blinded fashion. The *P*-values for assessing the number of LC3 dots/cell were determined using Student's *t*-test.

### Clearance of mutant A53 T α-synuclein

As previously described (54), stable inducible PC12 cell lines expressing A53 T α-synuclein were induced with 1 μg/ml doxycycline for 48 h. Transgene expression was switched off by removing the antibiotic from the medium and then the cells were treated for 24 h with the vehicle alone (DMSO), rapamycin (200 nm) or PPP at the indicated concentrations for a further 24 h. The levels of A53 T transgene were then assessed by western blot analysis. Experiments were performed in triplicate and on at least three different occasions and quantified by densitometry normalized to actin.

### Ovoalbumin degradation assay

HeLa cells were seeded in six-well plates and transiently transfected with 1 μg of a phagocytosis-competent, chimeric immunoglobulin receptor expression vector (Fcγ-RI-γ) (50) and cultured in full medium for 24 h. To allow phagocytosis of IgG-coated beads, HeLa cells were incubated (2 h; 37°C) with fluorescent beads (Polysciences) covalently conjugated with human polyclonal IgG and ovalbumin (OVA), thoroughly washed and then incubated for a further 24 h to permit degradation of internalized beads in the presence or absence of the anti-human IGF-1R monoclonal antibody (MAB391). The cells were then placed at 4°C to prevent bead internalization, stained with a human IgG-specific antibody (Jackson ImmunoResearch) to mark non-internalized beads and then fixed. Internalized beads were subsequently recovered by cell disruption (lysis with 1% Triton X-100 for 30 min followed by homogenization by passage through a hypodermic needle in the presence of proteases inhibitors) and then incubated with an OVA-specific FITC-conjugated antibody (Abcam) to detect remaining bead-associated OVA, which was quantified by flow cytometry on a FACSCalibur apparatus (Becton Dickinson), as previously reported (53).

### Transferrin uptake and internalization assay

In order to measure clathrin-dependent endocytosis, internalization assays of fluorochrome-conjugated human transferrin (Tf) were performed using Alexa 647-conjugated Tf (50 μg/ml) at 37°C for 0 up to 15 min as previously described (28). Briefly, HeLa cells were washed once with serum-free medium, trypsinized, collected in Eppendorf tubes, chilled on ice for 15 min to arrest endocytosis and finally loaded with transferrin to allow the binding to the tranferrin receptor (20 min on ice). After binding, the cells were shifted back to 37°C and the transferrin internalization chased at different time points up to 15 min. The amount of internalized ligands was measured by FACS analysis (FacsCalibur, Becton&Dickinson). The graphs report the total amount of internalized transferrin at different time points (as indicated), and normalized to the amount of transferrin bound to the cognate receptor at the cell surface at Time 0 (i.e. before the temperature shift).

### Immunofluorescence, live imaging and confocal microscopy

Cells grown on glass coverslips were fixed in 4% paraformaldehyde for 10 min and then permeabilized with either pre-chilled methanol (5 min at −20°C) or 0.1% Triton X-100 (for the phalloidin and GFP-ATG16L1/phalloidin co-staining). 4% goat serum (Sigma) in 1× PBS was used for blocking (2 h at room temperature) and for the incubation with the appropriate primary and secondary antibodies, when necessary. For the endogenous LC3 and LAMP-1 staining, the cells grown on glass coverslips were fixed and permeabilized with pre-chilled methanol (5 min at −20°C). Four percent goat serum (Sigma) in 1× PBS was used for blocking (2 h at room temperature) and for the incubation with the appropriate primary and secondary antibodies. Coverslips were left in the primary antibody overnight at 4°C. Secondary antibodies were Alexa Fluor-conjugated antibodies (Molecular Probes, Invitrogen). A Zeiss Axiovert 200 m microscope with a LSM510 confocal attachment (63 × NA 1.4 Plan-Apochromat oil-immersion lens LSM510 META, Carl Zeiss) along with the LSM510 Image analyser software (version 3.2, Carl Zeiss) was used for fluorescent confocal microscopy involving immunofluorescent staining with Alexa-Fluor-conjugated secondary antibodies or fluorescently tagged proteins. All confocal images were taken with a 63× oil-immersion lens. Microscopy was performed on cells fixed on coverslips. Coverslips were mounted in Prolong Gold Antifade reagent with 4′,6-diamidino-2-phenylindole (Molecular Probes, Invitrogen). ImageJ and Photoshop (Adobe) were used for further analysis and processing of confocal images.

For the pH determination of acidic organelles, LysoSensor Yellow/Blue DND-160, which produces blue fluorescence in a neutral environment but shifts to yellow fluorescence in more acidic compartments (p*K*_a_ ≈4.2), was used according to the manufacturer's instruction (Molecular Probes, Invitrogen). Briefly, cells were seeded on a MatTek Petri dish (MatTek, Ashland MA USA) at a density of ∼1.5 × 10^5^ cells per dish. The cells were treated or not for 24 h with MAB391, loaded with the LysoSensor tracer for 1 h at 37°C, washed twice with medium and imaged immediately at 37°C. At least 10 pictures of live cells were taken for each experimental condition. Live imaging was performed on an Axiovert 200 M microscope with a LSM 710 confocal attachment using a 63× 1.4 NA Plan Apochromat oil-immersion lens (Carl Zeiss). In particular, the cells were excited at 365 nm and images were taken at 450 and 510 nm of emission, respectively. ImageJ and Photoshop (Adobe) were used for the analysis and processing of confocal images.

#### *In vivo* experiments

### Maintenance of zebrafish stocks and collection of embryos

All zebrafish husbandry and experiments were performed in accordance with UK legislation under a license granted by the Home Office and with local ethical approval. Zebrafish were reared under standard conditions on a 14 h light/10 h dark cycle. Embryos were collected from natural spawnings, staged according to the established criteria (55) and reared in embryo medium (5 mm NaCl, 0.17 mm KCl, 0.33 mm CaCl_2_, 0.33 mm Mg_2_SO_4_, 5 mm HEPES).

### Determination of the maximum-tolerated concentration of compounds in larval zebrafish

Compound exposure experiments were performed on wild-type larvae (TL strain) from 2 to 3 days post-fertilization (d.p.f.). Concentration response assays were performed over log intervals, namely from 1 nm to 100 μm for PPP, in order to determine the maximum non-toxic concentration (MTC) for subsequent autophagy assay experiments (*n* = 30 larvae per concentration). The MTC for ammonium chloride (NH_4_Cl) was previously determined as 100 mm (39,40). Compound exposure experiments were performed in the dark at 28.5°C.

### Measuring endogenous LC3-II in larval zebrafish

LC3-II assays were performed at the following concentrations: ammonium chloride at 100 mm, PPP at 100 µm for 24 h. Wild-type larvae (*n* = 30 per treatment group) at 2 d.p.f. were exposed to PPP or embryo medium (untreated control) for 24 h with or without the addition of ammonium chloride (39). Larvae were then transferred to chilled tubes, homogenized in lysis buffer and finally processed for western blotting as described above.

### Aggregate analysis in the transgenic HD zebrafish

Aggregate counting was performed using the heterozygous larvae from the Tg(rho:EGFP-HTT71Q)^cu5^ zebrafish (41) (hereafter referred to as transgenic HD zebrafish). Embryos from out-crossed transgenic HD zebrafish were raised in 0.2 mm 1-phenyl-2-thiourea (PTU) from 1 to 3 d.p.f. in order to inhibit pigment formation, screened for transgene expression using EGFP fluorescence then washed twice in the embryo medium to remove PTU. From 3 to 7 d.p.f., transgenic HD zebrafish larvae were dark-reared in embryo medium alone or embryo medium contain containing either DMSO, 30 μm clonidine or 100 µm PPP. Embryo medium and compounds were replenished daily. At 7 d.p.f., larvae were anaesthetized by immersion in 0.2 mg/ml 3-amino benzoic acid ethylester (MS222), then fixed using 4% paraformaldehyde (PFA) in PBS at 4°C. Larvae were washed briefly in PBS, allowed to equilibrate in 30% sucrose in PBS then embedded in OCT medium (Tissue-Tek) and frozen on dry ice for subsequent cryosectioning. Sections were cut at 10 ? m thickness using a Leica CM3050 cryostat and mounted in 80% glycerol in PBS. The total number of GFP-positive aggregates were counted over 100 μm of the central retina, either side of the optic nerve using mean values were calculated (*n* = 4 fish (8 eyes) for each treatment group). Sections were viewed and representative images acquired using a GX Optical LED fluorescent microscope, GXCAM3.3 digital camera and GX Capture software.

### Mice experiments

All mice experiments complied with Home Office project and personal animal licenses. At the start of the study, C57BL/6 mice were aged 35–42 days. Mice were separated by gender and divided into two groups, each containing three mice. One group received no treatment (vehicle, DMSO), while the other group was intraperitoneally injected with PPP (dissolved in DMSO) at 20 mg/kg/24 h for 3 days (36). Following this, mice were sacrificed. The liver and muscle were dissected, frozen immediately after removal and finally homogenized using a Wheaton glass homogenizer at 4°C in lysis buffer (0.25 m Tris, 150 mm NaCl, 1% Triton) in the presence of protease/phosphatase inhibitor cocktail (Roche Scientific). Homogenates were then centrifuge at 14000 rpm for 30 min at 4°C, the supernatants were collected and analysed by western blot as described above.

### Statistical analysis

In all the experiments, we determined the significance levels for comparisons between groups by a two-tailed Student's *t*-test, repeated measurements or Factorial ANOVA test using STATVIEW v4.53 (Abacus Concepts), where appropriate. Densitometric analysis on the immunoblots was performed using Image J software. Experiments were performed at least three times in triplicate. In all the main or supplementary figures, error bars represent standard deviations. The *P*-values for assessing densitometric analysis on the immunoblots, luciferase assays, EGFP-HDQ74 aggregation, GFP-LC3 dots formation, GFP-ATG16L1 vesicles analysis and transferrin uptake assays were determined using Student's *t*-test.

## SUPPLEMENTARY MATERIAL

Supplementary Material is available at *HMG* online.

## FUNDING

Funding to pay the Open Access publication charges for this article was provided by the Wellcome Trust.

## Supplementary Material

Supplementary Data
