# Supplementary material for: IGF-1 receptor antagonism inhibits autophagy
Source: Hum Mol Genet. 2013 Jun 25;22(22):4528–44. doi: 10.1093/hmg/ddt300 (PMC3889807; doi:10.1093/hmg/ddt300)
Supplement: Supplementary Data [file supp_22_22_4528__index.html]

IGF-1 Receptor Antagonism Inhibits Autophagy — IGF-1 receptor antagonism inhibits autophagy — IGF-1 receptor antagonism inhibits autophagy — Supplementary Data 

# IGF-1 receptor antagonism inhibits autophagy

## 

Supplementary Data

**Files in this Data Supplement:**

- Supplementary Data - Pdf file
