# Supplementary material for: IGF-1 receptor antagonism inhibits autophagy
Source: Hum Mol Genet. 2013 Jun 25;22(22):4528–44. doi: 10.1093/hmg/ddt300 (PMC3889807; doi:10.1093/hmg/ddt300)
Supplement: Supplementary Data [file supp_ddt300_ddt300supp.pdf]

SUPPLEMENTARY MATERIAL

IGF-1 receptor antagonism inhibits autophagy

Maurizio Renna, Carla Figueira-Bento, Angeleen Fleming, Fiona M. Menzies, Farah H. Siddiqi, Brinda Ravikumar, Claudia Puri, Moises Garcia-Arencibia, Oana Sadiq, Silvia Corrochano, Sarah Carter, Steve DM. Brown, Abraham Acevedo-Arozena and David C. Rubinsztein

This file includes:

1. Supplementary Figures (Fig. S1-S6)
2. Supplementary Figure Legends
3. Supplementary Results
4. Supplementary Note
5. Supplementary References

1. SUPPLEMENTARY FIGURE S1

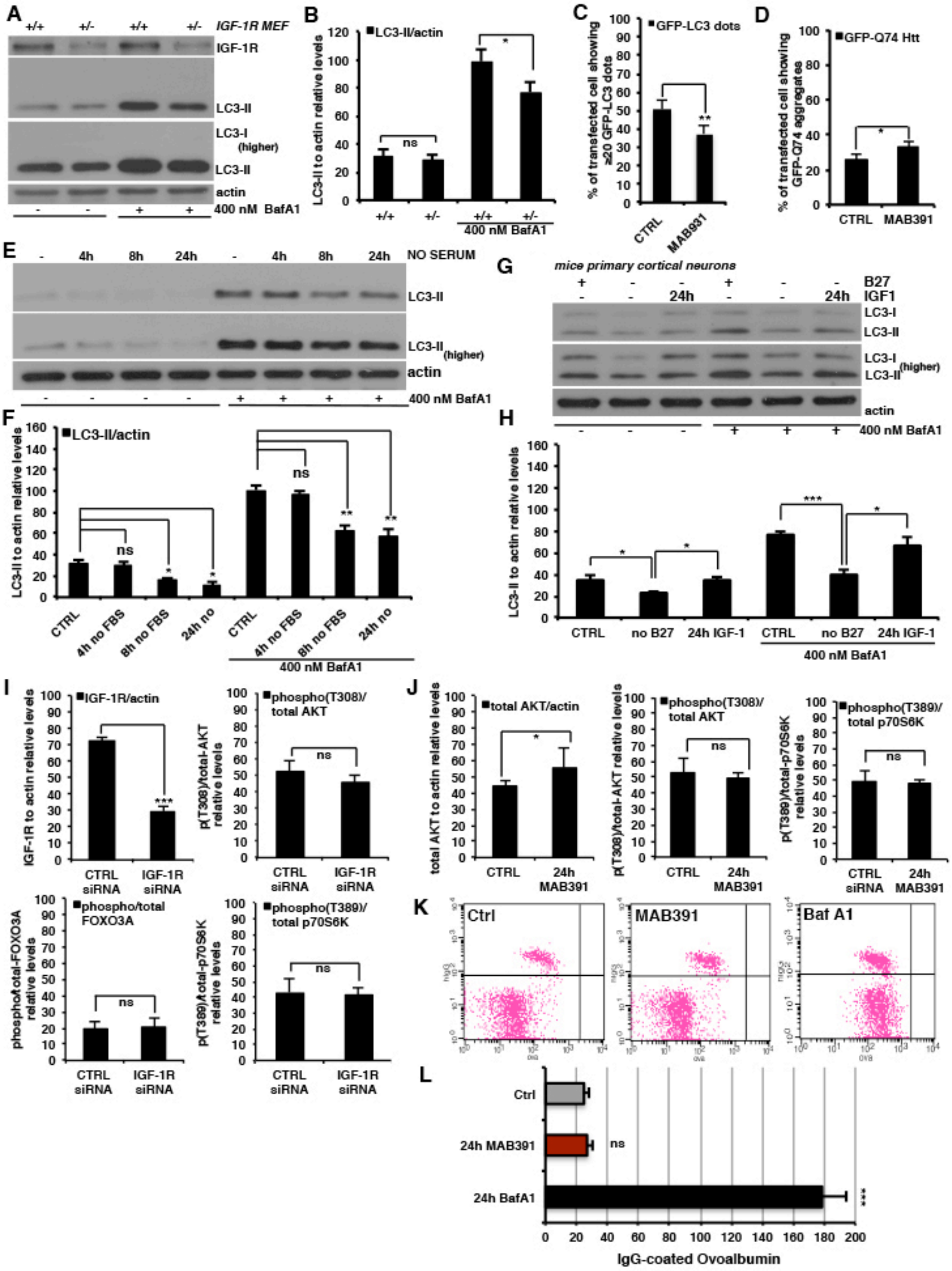

SUPPLEMENTARY FIGURE S2

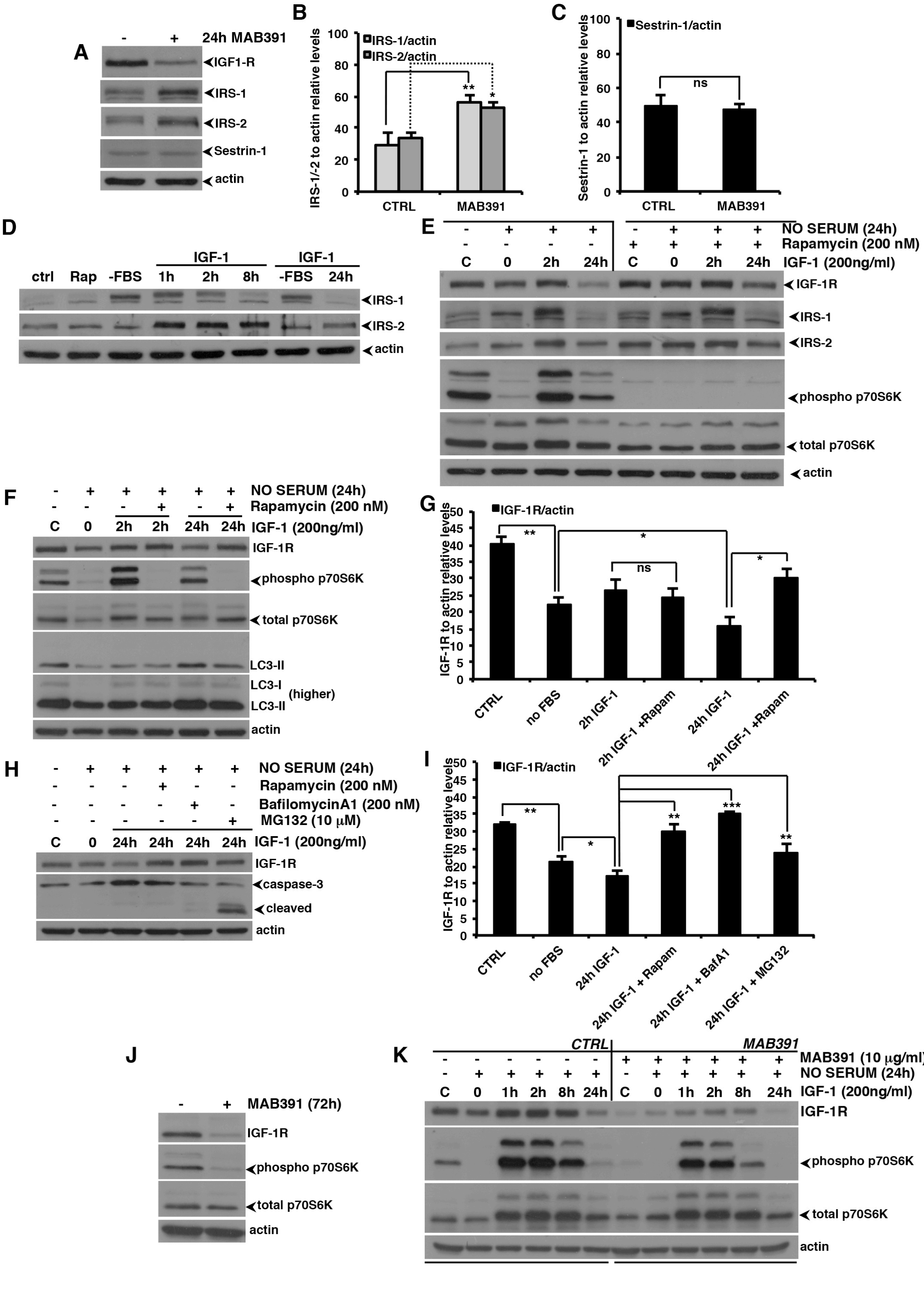

SUPPLEMENTARY FIGURE S3

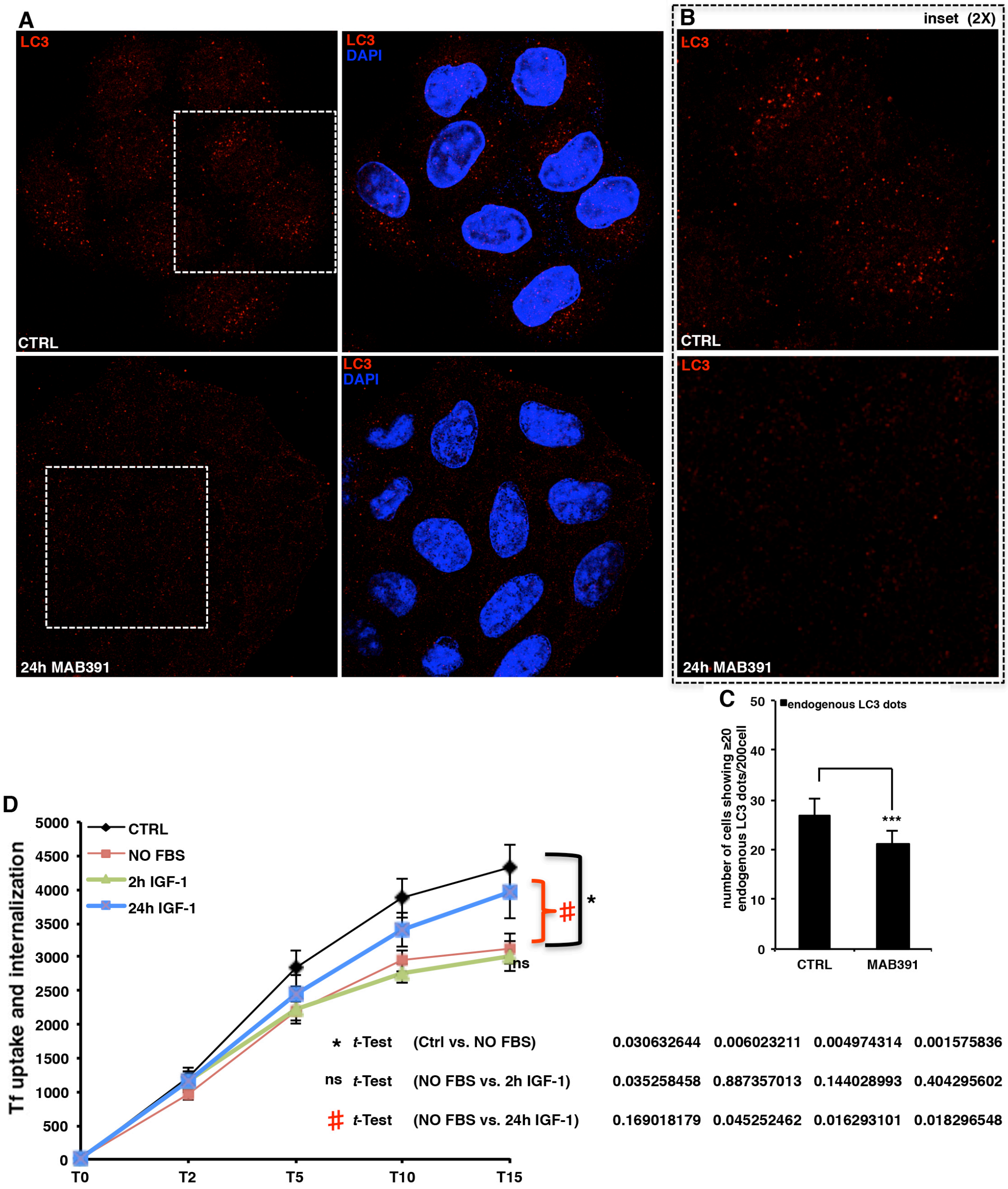

*	*t*-Test	(Ctrl vs. NO FBS)	0.030632644	0.006023211	0.004974314	0.001575836
ns	*t*-Test	(NO FBS vs. 2h IGF-1)	0.035258458	0.887357013	0.144028993	0.404295602
#	*t*-Test	(NO FBS vs. 24h IGF-1)	0.169018179	0.045252462	0.016293101	0.018296548

SUPPLEMENTARY FIGURE S4

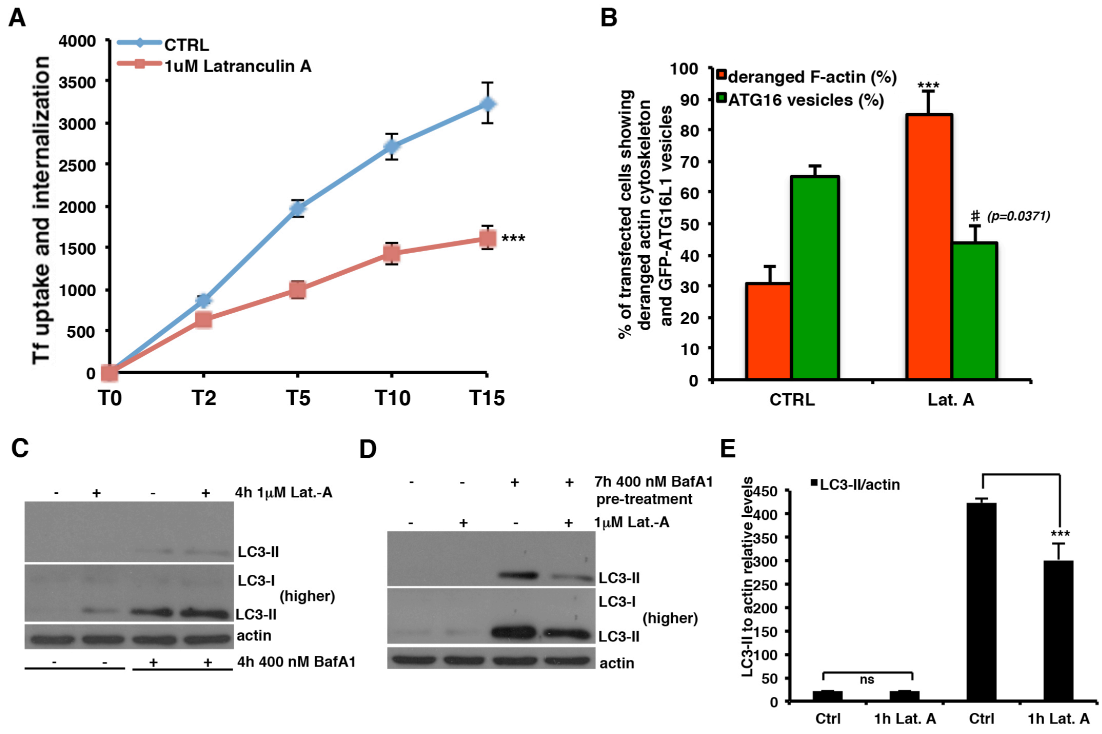

SUPPLEMENTARY FIGURE S5

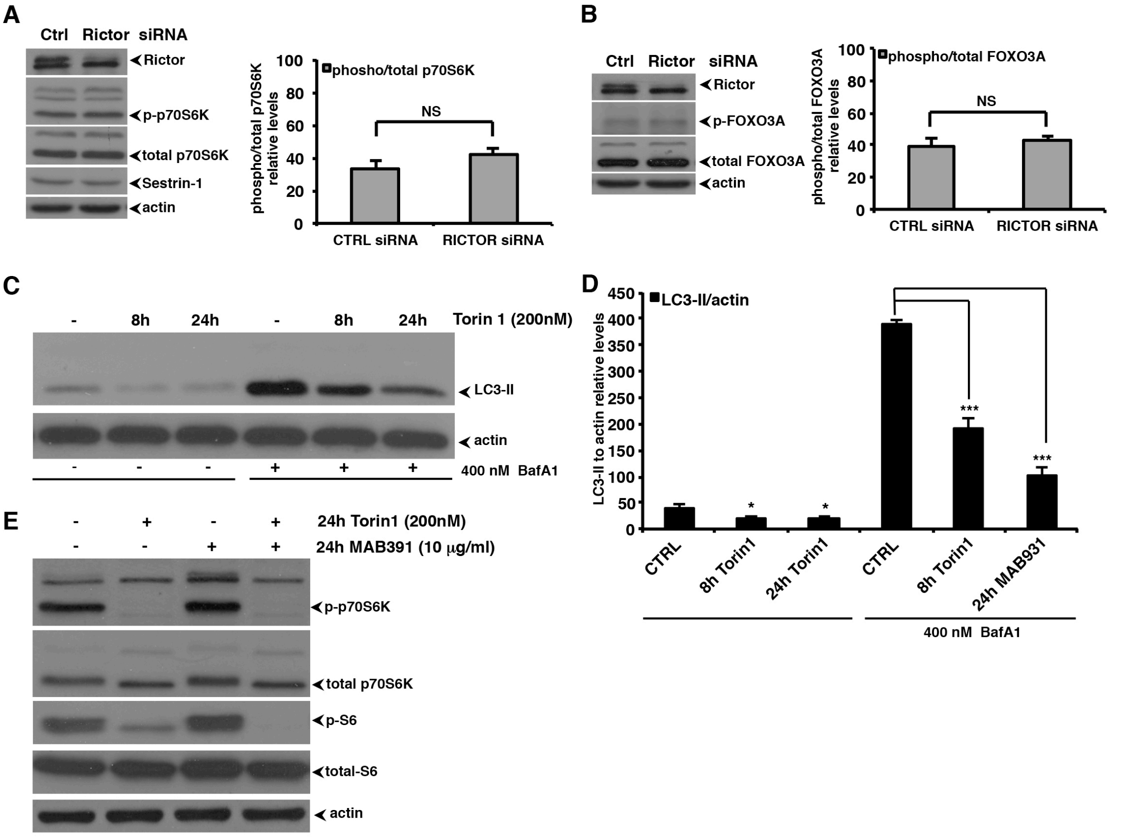

SUPPLEMENTARY FIGURE S6

Picropodophyllin (PPP)

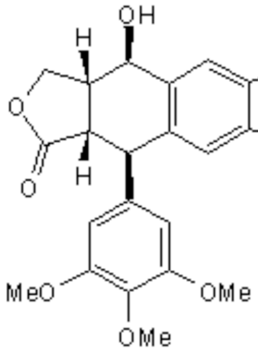

Alternative Name: Picropodophyllotoxin

Chemical Name: (5*R*,5a*S*,8a*R*,9*R*)-5,8,8a,9-Tetrahydro--9-hydroxy-5-(3,4,5-trimethoxyphenyl)-furo[3',4':6,7-]naphtho[2,3-*d*]-1,3-dioxol-6(5a*H*)-one

Orally active IGF1R selective inhibitor
No activity at the insulin receptor, FGFR, PDGFR or EGFR
Inhibits IGF1R autophosphorylation (IC50 ~ 1 nM)
Upregulates apoptosis
Antiproliferative effects in multiple cancer cell lines (IC_{50} = 0.05 - 15 μM) Anticancer and antineovascularization activity *in vivo*
LD_{50}>500 mg/kg in rodents

RANGE OF CONCENTRATIONS EMPLOYED TO INHIBIT IN VITRO AND IN VIVO THE IGF-1R ACTIVITY

Cell-based assays: 0.1- 1 μM

Zebrafish larvae: 100 μM

Mice: 20 mg/kg/24h PPDPT (IP injection)

2. SUPPLEMENTARY FIGURE LEGENDS

Figure S1 IGF-1R depletion reduces autophagic flux without apparently affecting the activity of the IGF-1/AKT/mTOR pathway

(**A-B**) LC3-II levels were assessed in IGF-1R$^{+/+}$ and IGF-1R$^{+/-}$ MEF cells treated or not with Bafilomycin A_1 (400 nM for 4 hours). The graph in B shows the quantitative analysis of LC3-II levels relative to actin (n=3; *, p < 0.05; ns=non-significant). (**C**) HeLa cells were transfected with 0.5 μg of the GFP-LC3 expression vector. After 24 hours, cells were treated for 24 hours with MAB391. Cells were finally fixed and analysed by fluorescence microscopy. The p value for assessing the number of GFP-LC3 dots was determined using Student's t-test (n=3; CTRL vs. MAB391, ***p*=0.0032). (**D**) HeLa cells were transfected for 48 hours with 2 mg of the GFP-HD74 expression vector and then treated for 24 hours with MAB391. The p value for assessing EGFP-HDQ74 aggregation was determined using Student's t-test (n=3; CTRL vs. MAB391, **p*=0.0294). (**E-F**) HeLa cells were serum-starved for the indicated time points. For the assessment of autophagy flux by LC3-II levels, Bafilomycin A_1 (400 nM) was added to the cells in the last 4 hours before harvesting. The western blot panels showing the effect of serum deprivation on LC3-II levels are representative of three independent experiments performed in triplicate. The graph in F shows the quantitative analysis of LC3-II levels relative to actin (n=3; *, p < 0.05; **, p < 0.01; ns=non-significant). Please, note that the serum deprivation (or B27, in the case of primary neuronal cultures) approach we have used in the experiments described in this figure as well as in figure S2 should not be considered as a standard starvation protocol (such as incubation with HBSS or EBSS medium), as the media contains L-glutamine which would promote autophagy. (**G-H**) mouse primary cortical neurons were isolated and *in vitro* differentiated as detailed in the methods section. After at least 7 days, cells were deprived of B27 supplement (which contains insulin) for 24 hours and stimulated by 200 ng/ml of IGF-1 R_3 for the following 24 hours. For the assessment of autophagy by LC3-II levels, Bafilomycin A_1 (400 nM) was added to the cells in the last 4 hours before harvesting (n=3; *, p < 0.05; ***, p < 0.001). (**I**) HeLa cells were transfected for 72 hours with 50 nM of either control or anti-IGF-1R siRNA. The western blot panels showing the effect of IGF-1R knockdown on the IGF-1/AKT/mTOR pathway are reported in the main Figure 1J. The graphs report the quantitative analysis of IGF-1R levels (relative to actin) and of the indicated phospho-proteins (relative to the respective total proteins). The p values were determined by Student's t-test. (**J**) HeLa cells were treated for 24 hours with MAB391. The graphs show the quantitative analysis of the indicated phospho-proteins, relative to the levels of the corresponding total proteins and of AKT levels relative to actin shown in Figure 1K. The p values of the densitometric analysis were determined by Student's t-test. In all the panels, error bars represent standard deviations. (**K-L**) IGF-1R depletion does not influence the degradative activity of lysosomal compartment. HeLa cells were transfected with the Fcγ-RI-γ expression vector for 24 hours and then treated or not with MAB391. The phagocytosis and degradation of IgG-coated,

fluorescent ovoalbumin (OVA) beads were then measured a detailed in the methods section. The rate of OVA degradation was not significantly affected in IGF1-R depleted cells (red bar), compared to control (gray bar). As an internal standard, control cells were treated with Baf-A_1, which greatly reduced OVA degradation (black bar). The *p* values for the rate of degradation of OVA-coated beads were determined using Student's t-test on three independent experiments performed in triplicate (n=3; ***P<0.0001).

Figure S2 IRS-1 and IRS-2 levels may regulate the signalling feedback loop

(**A-C**) HeLa cells were treated for 24 hours with MAB391. The western blot panels reporting the effect of MAB391 on IRS-1/-2 and sestrin-1 levels are representative of three independent experiments. The graphs report the quantitative analysis of IRS-1, IRS-2 and sestrin-1 relative to actin. The p values were determined by Student's t-test. (**D**) HeLa cells were serum-starved for 24 hours and then stimulated by IGF-1 R_3 for the indicated time points. The western blot panels are representative of at least three independent experiments performed in triplicate. (**E**) Vehicle (DMSO) or rapamycin pre-treated Hela cells (24 hours at 200 nM) were serum-starved for 24 hours and then stimulated by IGF-1 R_3 for either 2 or 24 hours. The western blot panels reporting the effect of serum deprivation/IGF-1 add-back protocol on the IGF-1R, IRS-1, IRS-2 and phospho/total p70S6Kinase levels are representative of three independent experiments performed in triplicate. (**F-G**) A mTOR-dependent feedback loop controls the IGF-1R levels upon sustained IGF-1 forward signalling. HeLa cells were serum-starved for 24 hours and then stimulated by IGF-1 R_3 for the indicated time points, in the presence or not of rapamycin. The western blot panels are representative of three independent experiments performed in triplicate (n=3; *, p < 0.05; **, p < 0.01). (**H-I**) the IGF-1R turnover is both lysosome- and UPS-dependent. HeLa cells were serum-starved for 24 hours and then stimulated by IGF-1 R_3 for 24 hours, in the presence or not of rapamycin, Bafilomycin A_1 or the proteasome inhibitor MG132. The western blot panels are representative of three independent experiments performed in triplicate (n=3; *, p < 0.05; **, p < 0.01). In all the panels, error bars represent standard deviations. (**J-K**) HeLa cells were pre-treated for 24 hours with the anti IGF-1R neutralizing antibody (MAB391), serum-starved for the following 24 hours and finally stimulated by 200 ng/ml of IGF-1 R_3 analogue for the indicated time points. The western blot panels in J show the effect of long-term IGF-1R neutralization on IGF-1R and phospho/total p70S6K levels. The IGF-1R pre-neutralization does attenuate the PI3K/AKT/mTOR pathway activation in response to IGF-1R stimulation, but has discernable effect on the kinetics of the process.

Figure S3 IGF-1R depletion decreases number of autophagosomes

(**A-B**) HeLa cells seeded on glass coverslips were treated or not with the MAB391 neutralizing antibody. After 24 hours, cells were fixed, permeabilized in methanol, stained with anti-LC3 specific antibody, and finally analysed by confocal microscope. 2× magnifications of the red channel (insets) are shown in B. (**C**) Two hundred cells per experimental condition were analysed using a fluorescence microscope and the

number of endogenous LC3 dots were scored. The *p* value for assessing the number of endogenous LC3 dots/cell was determined using Student's t-test (n=3; ****P* <0.001). (**D**) Sustained IGF-1 forward signaling restores the inhibitory effects of serum-starvation on endocytosis. HeLa cells were serum-starved for 24 hours and then treated or not with 200 ng/ml of IGF-1 R_3 analogue for the indicated time points. The amount of internalized ligand was measured by FACS analysis. The *p* values to assess differences in the rate of internalization of transferrin receptor were determined by Student's t-test on six independent experiment (n=6; *p<0.05; ns= non significant). In all the panels, error bars represent standard deviations.

Figure S4 Effect of actin cytoskeleton depolymerization on autophagy

(**A**) HeLa cells were treated or not with 1μM of Latrunculin A for 1 hour. The binding and internalization assays of the fluorochrome-conjugated human transferrin were then performed. The amount of internalized ligand was measured by FACS analysis. The *p* values to assess differences in the rate of internalization of transferrin receptor were determined by Student's t-test on three independent experiment (n=3; ***p<0.001). (**B**) HeLa cells seeded on glass coverslips were transfected with 0.5 mg of the GFP-ATG16L1 vector. After 24 hours, cells were treated or not with 1mM of Latrunculin A for 1 hour, fixed, permeabilized, stained with phalloidin and finally analysed by confocal microscope. The *p* values for assessing the percentage of cells showing GFP-Atg16 vesicles/actin cytoskeleton derangement were determined using Student's t-test (n=3; # p<0.05; *** p<0.001). (**C**) Prolonged actin cytoskeleton depolymerization impairs autophagosome-lysosome fusion (1). HeLa cells were simultaneously treated with 1mM of Latrunculin A for 4 hours in the presence or not of Bafilomycin A_1 (400 nM). The western blot panels are representative of three independent experiments performed in triplicate. (**D-E**) To overcome the confounding effect of Latrunculin A on autophagosome-lysosome fusion, HeLa cells were pre-treated with Bafilomycin A_1 for 7 hours, and then Latrunculin A (or DMSO) was added for 1 hour before harvest. The graph in E shows the quantitative analysis of LC3-II levels relative to actin. In all the panels, error bars represent standard deviations. The *p* values were determined by Factorial Anova (n=3; *** $p < 0.001$).

Figure S5 Rictor knock-down does not affect mTORC1 activity

(A-B) HeLa cells were transfected with 50 nM of either control or Rictor siRNA. The western blot panels showing the effect of Rictor knockdown on the IGF-1/AKT/mTOR pathway are representative of at least three independent experiments performed in triplicate. The *p* values were determined by Student's t-test on six independent experiment (n=3; ns=not significant). (**C-E)** Global mTOR inhibition reduces autophagosomes formation. HeLa cells were treated or not for the indicated time points with the pan mTOR catalytic inhibitor Torin1 at 200 nM. For the assessment of autophagy by LC3-II levels, Bafilomycin A_1 was added to the cells in the last 4 hours before harvesting. The western blot panels are representative of at least three independent experiments performed in triplicate. The graph in D shows the LC3-II levels relative to actin (n=3; * p <0.05; n=3; *** $p < 0.001$). In all the panels, error bars represent standard deviations. Representative blots showing the effect of

Torin1 treatment on known mTOR substrates (i.e.: phosphorylation of p70S6Kinase and S6 ribosomal protein, respectively) are reported in E.

Figure S6 Picropodophyllin (PPP) toxicity assessment
The orally active IGF-1R selective inhibitor Picropodophyllin (PPP) was dissolved in DMSO and added to normally growing HeLa cells for 24 hours at the indicated dosages. The definition of toxicity curves in larval zebrafish models and administration of PPP to mice for the in vivo experiments are detailed in the experimental procedures section.

3. SUPPLEMENTARY RESULTS

IRS-1 and IRS-2 levels may regulate the signaling feedback loop when IGF-1R is inhibited

A feedback loop effect has been described in cells that have mTOR hyperactivity. In these cells, the increased mTOR activity effectively reduces the levels of IRS-2, which, in turn, serves to decrease insulin-related signalling and tends to decrease mTOR activity (2). Consistent with this feedback mechanism, IRS-1 and IRS-2 levels increased after lowering IGF-1R levels in cell culture (Figure S2, A-B). Such a feedback mechanism regulated by IRS-1/-2 would be compatible with our observation that IGF-1 stimulation of serum-starved cells results in a rise and fall of mTOR activity that is paralleled by similar changes in AKT activity (Figure 2, A-B), suggesting that the feedback acts upstream of the AKT module. We also examined the levels of another feedback system that has been recently described to regulate mTOR via the protein sestrin-1 (3), but did not observe any change in our experimental system (Figure S2, A and C).

Notably, IRS-1 and IRS-2 levels were also increased after 24 h serum starvation compared to cells kept in full medium, rose transiently after 2h IGF-1 administration and then fell again after 24 hours of IGF-1 treatment (Figure S2D), in parallel with the changes observed in AKT/p70S6K phosphorylation (see main Figure 2, A-B). Moreover, the changes in IRS-1 and IRS-2 levels in this paradigm were m-TOR dependent, as the IRS-2 alterations, in particular, were largely obliterated by pre-treatment with the mTORC1 inhibitor rapamycin, which also increased IRS-2 levels at all times, compatible with the feedback effect being regulated by mTOR (Supplementary Figure S2, E). Since increased IRS-1/-2 levels will enhance AKT activation and mTOR activity in turn, this might explain why decreasing IGF1-R levels for a prolonged period (such as 24 hours) does not obviously reduce p70S6K activity. Interestingly, we also observed that levels of IGF-1R itself decreased after prolonged IGF-1 exposure in an mTOR-dependent fashion, since the decrease was antagonised by rapamycin pre-treatment (Figure S2, F-G), suggesting therefore that these changes in IGF1-R levels were mTORC1-dependent. We also showed by using proteasome or lysosomal inhibitors, that the IGF-1R turnover is both lysosome- and UPS-dependent (Figure S2 H-I). Thus, an mTOR-dependent feedback loop controls the IGF-1R levels upon sustained IGF-1 forward signaling. However, these changes in IGF-1R levels are not sufficient to account for the signaling loop, since while prolonged treatment (up to 72 instead of 24 hours) with the MAB391 neutralizing antibody decreased both the steady state (Figure S2, J) and the peak of mTOR activity upon 2 hours of IGF-1 stimulation (Figure S2, K), it did not have any discernable effect on the kinetics of the process, nor the complete lack of mTOR activity at 24h (Figure S2, K). Thus, compensatory feedback loops constrain mTOR activity changes even when the levels of IGF-1R are reduced, providing a plausible explanation to our observations.

4. SUPPLEMENTARY NOTE

In a recently published article, Back et al. have proposed a role for Rictor in autophagy (4). In particular, they show that resveratrol induces senescence, and down-regulates the levels of Rictor, leading to decreased RhoA-GTPase activity and altered actin cytoskeleton organization. This resveratrol-induced premature senescence is associated with a blockade of autolysosome formation. Furthermore, exogenous over-expression of Rictor restores RhoA-GTPase activity and actin cytoskeleton network, and decreases resveratrol-induced senescence. It is noteworthy to say that the authors have not examined the effect of reducing Rictor/mTORC2 activity on autophagy. The experiments are confined to Rictor over-expression in resveratrol-treated cells, and do not examine thoroughly autophagosome formation (neither analysing the formation of pre-autophagosomal structures nor measuring properly the autophagic flux). Furthermore, the results shown could be due to other perturbations to the pathway operated by resveratrol and are rather reminiscent and suggestive of an impaired autophagosome-lysosome fusion, a later step in the autophagosome itinerary that requires an intact and functional actin cytoskeleton (1) (please, see also Figure S4, C).

5. SUPPLEMENTARY REFERENCES

(1) Lee JY, Koga H, Kawaguchi Y, Tang W, Wong E et al. (2010). HDAC6 controls autophagosome maturation essential for ubiquitin-selective quality-control autophagy. EMBO J. 29: 969-80.

(2) Shah OJ, Wang Z, Hunter T (2004). Inappropriate activation of the TSC/Rheb/mTOR/S6K cassette induces IRS1/2 depletion, insulin resistance, and cell survival deficiencies. Curr. Biol. 14, 1650-1656.

(3) Lee JH, Budanov AV, Park EJ, Birse R, Kim TE et al. (2010). Sestrin as a feedback inhibitor of TOR that prevents age-related pathologies. Science 327: 1223-1228.

(4) Back JH, Zhu Y, Calabro A, Queenan C, Kim AS et al. (2012) Resveratrol-Mediated Downregulation of Rictor Attenuates Autophagic Process and Suppresses UV-Induced Skin Carcinogenesis. Photochem. Photobiol. 88(5): 1165-1172.
